# Supplementary material for: Elucidation of the Fanconi Anemia Protein Network in Meiosis and Its Function in the Regulation of Histone Modifications
Source: Cell Rep. Author manuscript; Available in PMC 2016 Nov 4. (PMC5095620; doi:10.1016/j.celrep.2016.09.073)
Supplement: 1 [file NIHMS820305-supplement-1.pdf]

Cell Reports, Volume *17*

Supplemental Information

Elucidation of the Fanconi Anemia Protein Network in Meiosis and Its Function in the Regulation of Histone Modifications

Kris G. Alavattam, Yasuko Kato, Ho-Su Sin, So Maezawa, Ian J. Kowalski, Fan Zhang, Qishen Pang, Paul R. Andreassen, and Satoshi H. Namekawa

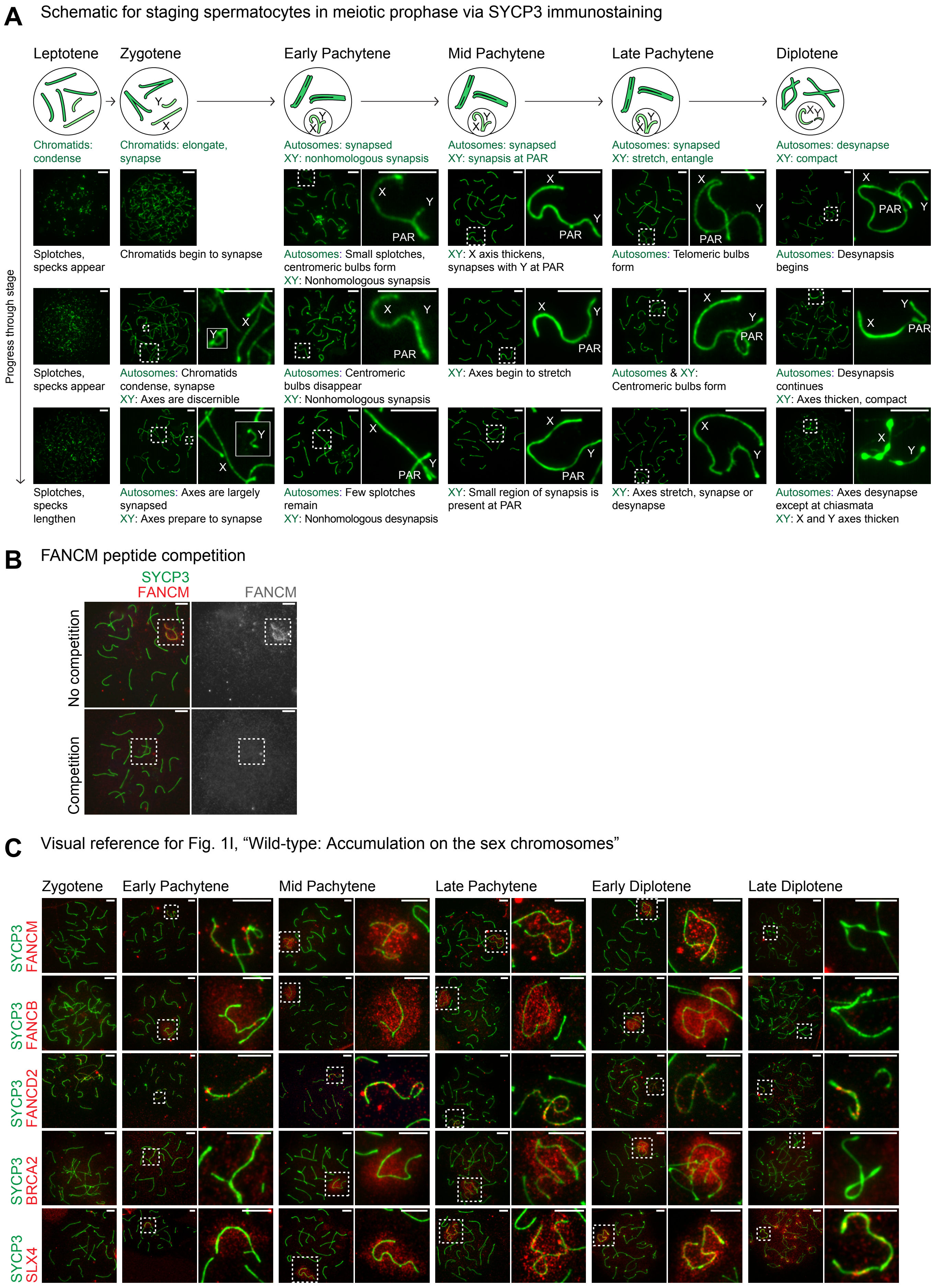

(legend on next page)

Figure S1. Cytology and criteria for staging spermatocytes in meiotic prophase via SYCP3 staining, related to Figure 1.

(**A**) Meiotic prophase is divided into four stages based on the presentation and pairing—or synapsis—of paternal and maternal chromatids, known as non-sister chromatids. Chromatids are the threadlike strands that chromosomes condense into during cell division. Essential to this condensation and synapsis is SYCP3 (shown in green), a structural component of the synaptonemal complex protein polymer. Through immunofluorescent microscopy of chromosome spreads stained with anti-SYCP3 antibody, it is possible to stage spermatocytes in meiotic prophase with a high degree of accuracy. In the first stage of meiotic prophase, the leptotene stage, maternal and paternal chromatids begin to condense and elongate. In the following stage, the zygotene stage, the chromatids continue to elongate and begin to synapse. The subsequent pachytene stage lasts the longest and, as such, is subdivided into three stages to account for its many appearances: the early, mid, and late pachytene stages. In the early pachytene stage, all chromosomes that are not sex chromosomes—that is, autosomes—have synapsed, and the male sex chromosomes, X and Y, undergo partial synapsis at a small region known as the pseudo-autosomal region (PAR). During the early pachytene stage, XY synapsis increases until most of the Y chromosome axis is nonhomologously synapsed to X; then, as spermatocytes progress through the mid and late pachytene stages, X and Y desynapse. In the next stage of meiotic prophase, the diplotene stage, X and Y compact while the autosomes progressively desynapse except at specialized regions of contact known as chiasmata, where the exchange—or recombination—of genetic material occurs between non-sister chromatids. In the panel, the progression through stages are shown top to bottom and are indicated by an arrow: top images in each column represent early examples of each stage, middle images represent intermediate examples, and bottom images represent late examples. The sex chromosomes are indicated by dashed squares, and these squares are magnified in panels to the right. In the sample cytology for the zygotene stage, magnified images of Y are inlaid with magnified images of X. X: X chromosome; Y: Y chromosome; PAR: pseudo-autosomal region. Scale bars: 5 µm.

(**B**) Immunostains using anti-FANCM and -SYCP3 antibodies in meiotic chromosome spreads from wild-type mice with and without FANCM peptide competition. Sex chromosomes are indicated by dashed squares. Consistent results were obtained with n = 3 independent wild-type mice. Scale bars: 5 µm.

(**C**) Representative immunostains of meiotic chromosome spreads in different stages of meiotic prophase using anti-FANCM, -FANCB, -FANCD2, -BRCA2, and -SLX4 antibodies as designated to the left of rows. Stages of meiotic prophase are labeled above columns. Scale bars: 5 µm.

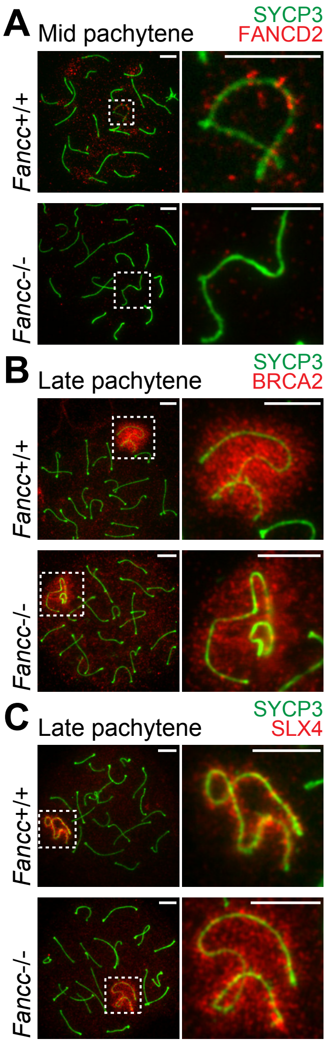

D *Fancc*-/-: Accumulation on the sex chromosomes

	EP	MP	LP	ED	LD
FANCD2					
BRCA2		domain	domain	domain	
SLX4	domain	domain	domain	domain	axial

(green) present
(white) absent

Figure S2. Function of FANCC in the FA-BRCA pathway during meiosis, related to Figure 2.

(**A-C**) Immunostains using indicated antibodies in meiotic chromosome spreads from *Fancc*-/- mice and wild-type littermate controls. Stages are labeled above, genotypes are labeled to the left. Dashed squares border sex chromosomes and are magnified to the right. Consistent results were obtained with n = 3 independent littermate pairs. Scale bars: 5 µm.

(**D**) Summary of temporal and spatial localization of anti-FA protein antibodies on the sex chromosomes in *Fancc*-/- mice; summaries of localization in wild-type mice are shown in Figures 1I and S1C.

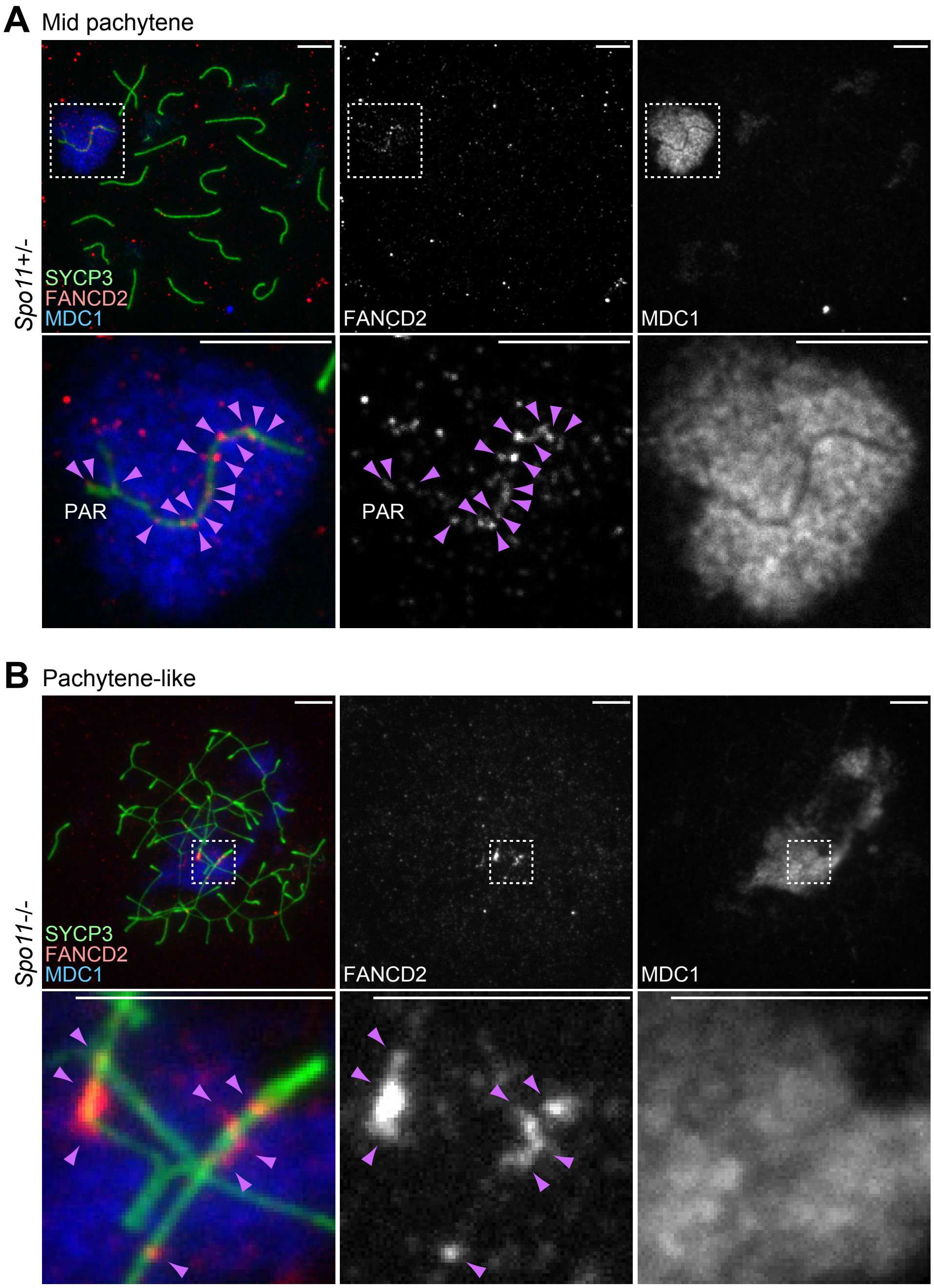

Figure S3. FANCD2 foci are present in the chromatin domain with meiotic silencing independent of SPO11-generated DSBs, related to Figure 5.
(**A, B**) Immunostains using indicated antibodies in meiotic chromosome spreads from *Spo11*-/- mice and control littermates. Stages are labeled above, genotypes are labeled to the left. Dashed boxes border selected nuclear regions and are magnified below. Arrowheads: colocalization of FANCD2 and MDC1. Consistent results were obtained with n = 3 independent littermate pairs. PAR: pseudo-autosomal region. Scale bars: 5 µm.

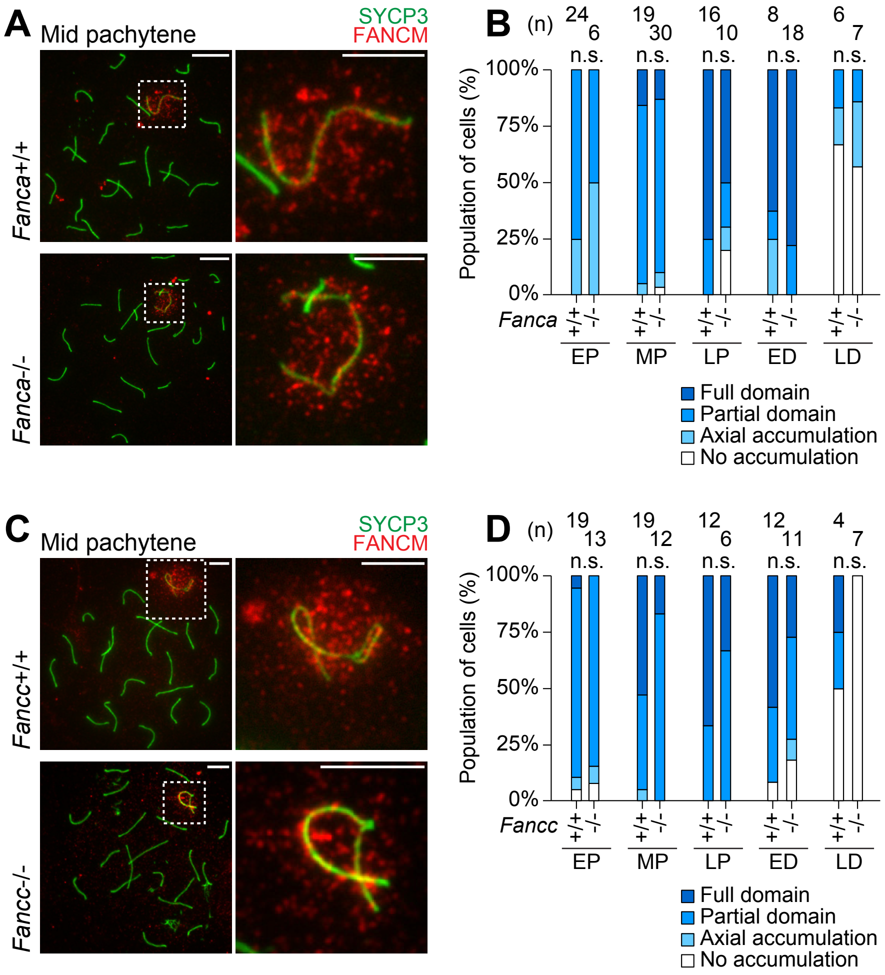

Figure S4. The FA core complex is dispensable for the regulation of FANCM, related to Figure 6.

(**A, C**) Immunostains using indicated antibodies in meiotic chromosome spreads from *Fanca*-/- (**A**) and *Fancc*-/- (**C**) mice, and corresponding wild-type littermate controls. Stages are labeled above, genotypes are labeled to the left. Dashed squares border sex chromosomes and are magnified to the right. Consistent results were obtained with n = 4 independent littermate pairs for each model. Scale bars: 5 µm.

(**B, D**) Categorical staining patterns for FANCM accumulation on sex chromosomes of *Fanca* (**B**) and *Fancc* (**D**) spermatocytes. Numbers of spermatocytes analyzed are noted above each graph. Accumulation was scored according to criteria described in the legend for Figure 4D. Data are aggregated from n = 3 littermate pairs for each model. p values are derived from Pearson's chi-square tests: n.s.: not significant.

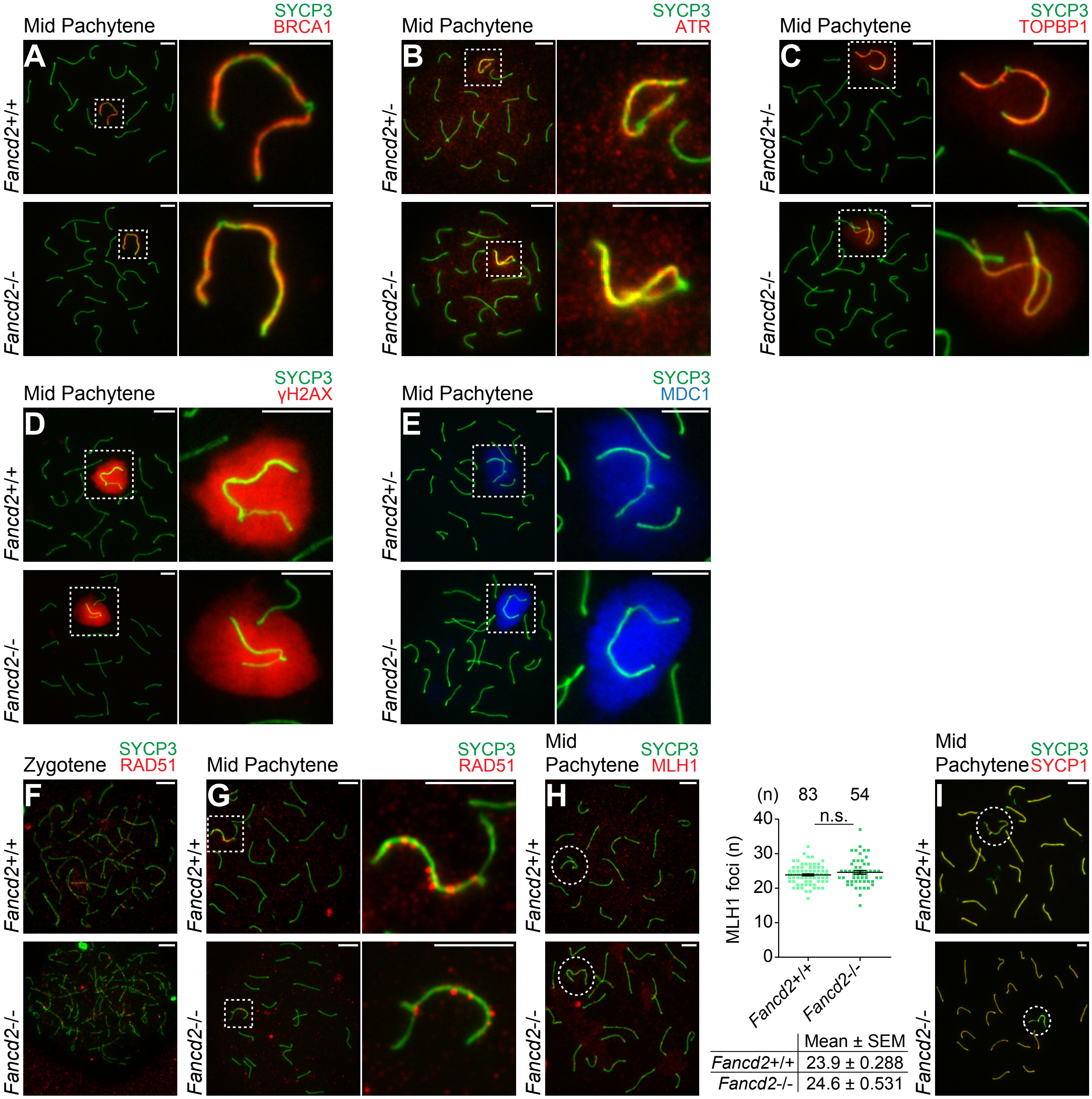

Figure S5. Regulation of DDR and recombination factors on the sex chromosomes in *Fancd2* mutant mice, related to Figure 6.

(**A-I**) Immunostains using the indicated antibodies in meiotic chromosome spreads from *Fancd2*-/- mice and wild-type or heterozygous littermate controls. Stages are labeled above, genotypes are labeled to the left. Dashed squares border sex chromosomes and are magnified to the right. Dashed circles border the sex chromosomes in (**H, I**). Consistent results were obtained with n = 3 independent littermate pairs. Scale bars: 5 μm.

(**H**) Bars represent means and SEMs. Mean ± SEM for each genotype is shown below. Data are aggregated from n = 3 littermate pairs of wild-type and mutant *Fancd2* mice. p values are derived from an unpaired, two-tailed Student's t test: n.s.: not significant, p > 0.05.

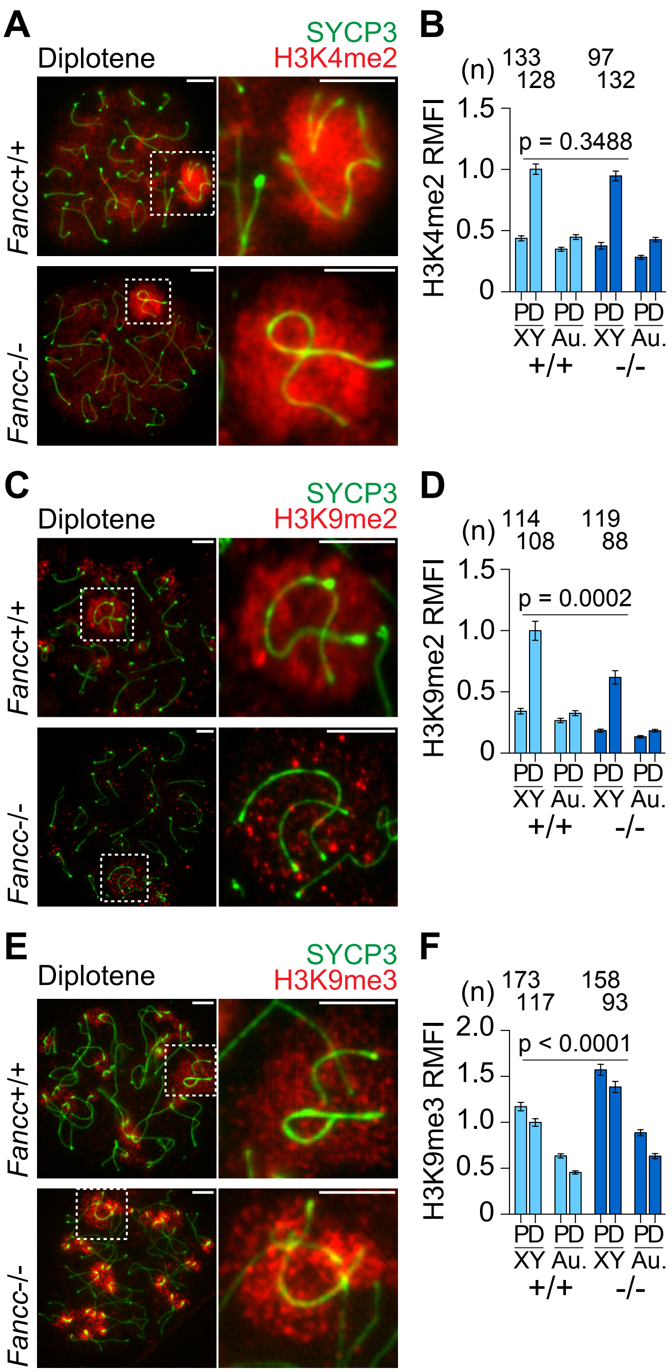

Figure S6. FANCC regulates H3K9 methylation, related to Figure 7.

(**A, C, E**) Immunostains using the indicated antibodies in meiotic chromosome spreads from *Fancc*-/- mice and corresponding wild-type controls. Stages are labeled above, genotypes are labeled to the left. Dashed squares border sex chromosomes and are magnified to the right. Scale bars: 5 µm.

(**B, D, F**) Quantification of H3K4me2 (**B**), H3K9me2 (**D**), and H3K9me3 (**F**) relative mean fluorescence intensity (RMFI) on sex chromosomes (XY) and autosome regions (Au.) in pachytene (P) and diplotene (D) spermatocytes. Numbers of spermatocytes analyzed are noted above each graph. Bars represent means and SEMs. Data are aggregated from n = 3 independent littermate pairs of wild-type and *Fancc*-/- mice. p values, indicated in the panels, are derived from one-way ANOVA and Tukey's method posttests.

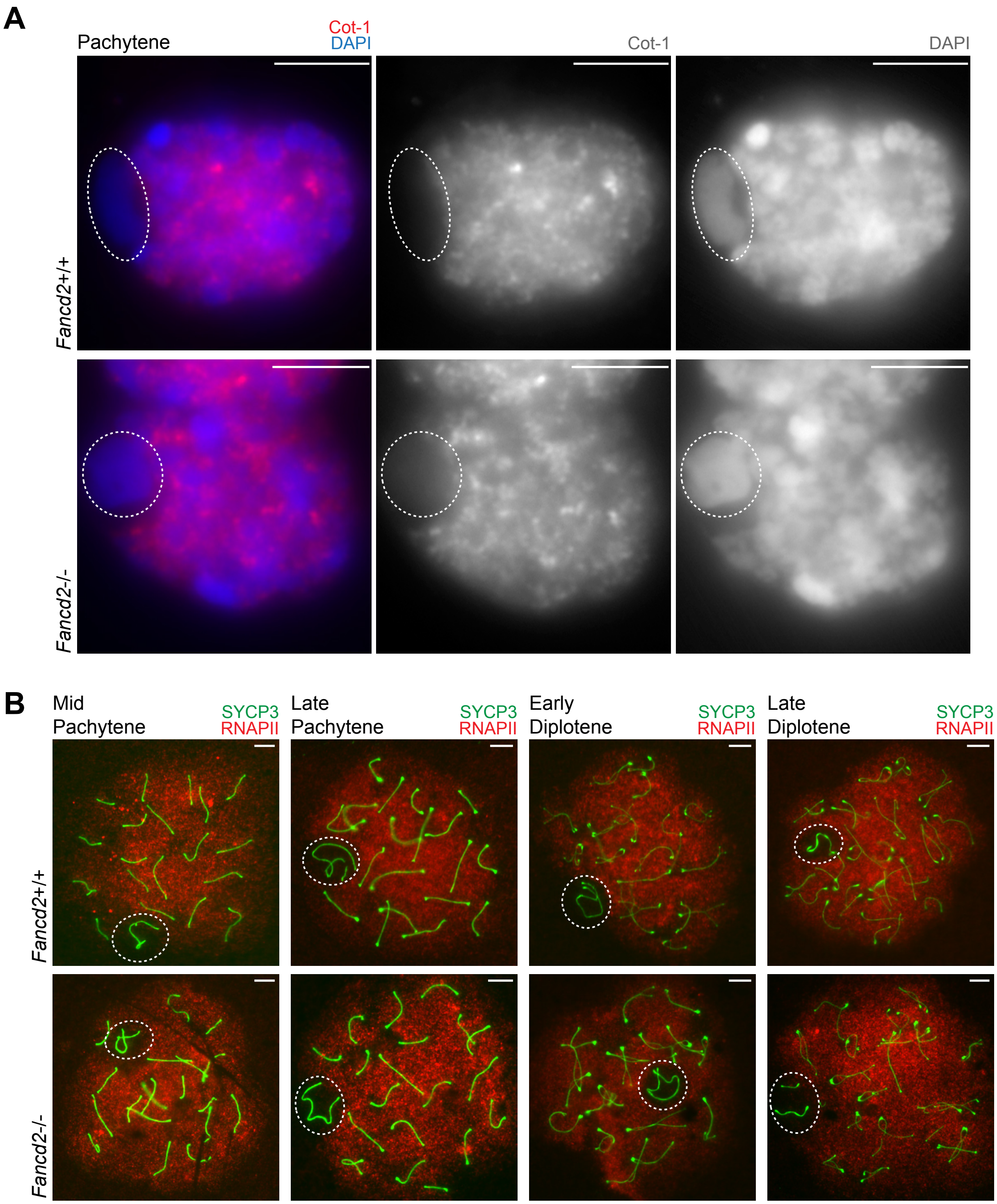

Figure S7. Large-scale detection of transcription remains unchanged in *Fancd2* mutants, related to Figure 7.

(**A**) Cot-1 RNA FISH (red signal) and DAPI (blue signal) in pachytene spermatocytes from *Fancd2*-/- and wild-type littermate control slides that maintain the three-dimensional architecture of nuclei (3D slides).

(**B**) Immunostaining of meiotic chromosome spreads using the indicated antibodies in *Fancd2*-/- mice and wild-type littermate controls.

(**A, B**) Consistent results were obtained with n = 3 independent littermate pairs of mice. Dashed circles border and denote the XY chromatin. Scale bars: 5 µm.

Supplemental Experimental Procedures

Generation of mice with conditional deletion of *Brca1* exon 11

Mice with conditional deletion of *Brca1* exon 11 using *Ddx4*-cre were previously described (Broering et al., 2014). Briefly, mice with floxed alleles for *Brca1* exon 11 (Xu et al., 1999) were obtained from the National Cancer Institute mouse repository. *Ddx4*-cre (also known as *Vasa*-cre) transgenic mice (Gallardo et al., 2007) were obtained from the Jackson Laboratory. Because the *Ddx4*-cre allele needs to be transmitted from the paternal allele to generate mice with a germline-specific conditional deletion, males with *Brca1*F/+ *Ddx4*-cre were mated with females homozygous for the floxed allele of *Brca1* exon 11 (*Brca1*F/F), and the conditional deletion model *Brca1*F/Δ *Ddx4*-cre (*Brca1*cKO) was obtained. We used *Brca1*F/+ *Ddx4*-cre littermates as controls.

Preparation and immunofluorescence of surface spreads of meiotic chromosomes

Meiotic chromosomes were analyzed with surface spreads prepared via hypotonic treatment, modified from an established protocol (Peters et al., 1997). Briefly, testes were excised and placed in PBS after removing extratesticular tissues. Seminiferous tubules (approximately one-quarter of an adult wild-type or heterozygous testis, and approximately one-half of an adult mutant testis) were transferred to a four-well dish (e.g., Thermo Scientific Nunc 4-Well Dishes, 144444) on ice. Three of the four wells contained 1 mL PBS, while the fourth well contained 1mL hypotonic extraction buffer (HEB, prepared as described in Peters et al.). In the first well containing 1 mL PBS, seminiferous tubules were gently unraveled into small clumps with fine-point tweezers, and care was taken not to tear or mince the seminiferous tubules. The clumps of seminiferous tubules were transferred to the second and third wells of 1 mL PBS for additional unraveling before transfer to the fourth well containing 1mL HEB. Once there, fine-point tweezers were used to carefully expose tubule surface area to HEB. The seminiferous tubules were incubated in HEB on ice for approximately three hours with gentle stirring every 30-45 minutes. After incubation, a small clump of seminiferous tubules—approximately four-to-six seminiferous tubules—was gently pulled and mashed between the tips of tweezers in 30 μL of sucrose (100 mM, pH 8.2) on a plain, uncharged microscope slide (e.g., Thermo Scientific Gold Seal, 3010-002). After approximately 15-25 mashes, a semi translucent cell suspension was formed. An additional 30 μL of sucrose was mixed with the suspension, gently pipetting up and down to mix and dilute the cell suspension. 30 μL volumes of the diluted cell suspension were applied to a positively charged slide (e.g., Thermo Scientific Probe On Plus, 22-230-900) that was incubated in chilled fixation solution (2% paraformaldehyde, 0.05% Triton X-100, and 0.02% sodium monododecyl sulfate, adjusted to pH 9.2 with sodium borate buffer, prepared as described in Peters et al.) for a minimum of two minutes. After applying the cell suspension/sucrose mixture, the slide was slowly, gently tilted up and down at slight angles to mix the cell suspension/sucrose mixture with remaining fixation solution. The previous steps were repeated until a desired number of slides were created. The slides were placed in closed humid chambers at room temperature for a minimum of one hour (maximum overnight) before opening the humid chamber lid to facilitate drying of the slides (approximately two hours). Once dry, the slides were washed in a low-concentration surfactant, 0.4% Photo-Flo 200 (Kodak, 146-4510), at room temperature two times for two minutes each. Slides were dried completely at room temperature (approximately 30 minutes) before staining or storage in slide boxes at -80°C.

For immunostaining experiments, surface spreads were incubated in PBST for 5-30 minutes before blocking in antibody dilution buffer (0.15% BSA, 0.1% Tween 20 in PBS), or 1% BSA dissolved in ddH2O, for an additional 30-60 minutes. Primary antibodies were diluted in antibody dilution buffer. Then, surface spreads were coated with 100 μL of the antibody solution, gently covered with Parafilm, and stored for a minimum of six hours (maximum overnight) in a

humid chamber at room temperature or 4°C. The following antibodies were used in this study: rabbit polyclonal anti-ATR (Cell Signaling, 2790), 1:50; rabbit polyclonal anti-BRCA1, 1:1500 (generated in the Namekawa lab (Ichijima et al., 2011)); rabbit polyclonal anti-BRCA2 (generated in the Andreassen lab in rabbits by fusing the 2800-3000 amino acid fragment of human BRCA2 to GST), 1:100; rabbit polyclonal anti-FANCB, 1:100 (generated in the Namekawa lab (Kato et al., 2015)); rabbit polyclonal anti-FANCD2 (E35), 1:200; rabbit polyclonal anti-FANCD2 (Novus, NB100-182), 1:200; rabbit polyclonal anti-FANCM (Fanconi Anemia Research Foundation, D3823), 1:100; rabbit polyclonal anti-H3K4me2 (EMD Millipore, 07-030), 1:500; mouse monoclonal anti-H3K9me2 (EMD Millipore, 07-441); rabbit polyclonal anti-H3K9me3 (EMD Millipore, 07-442), 1:250; rabbit polyclonal anti-RAD51 (Santa Cruz Biotechnology, sc-8349), 1:50; sheep polyclonal anti-MDC1 (Bio-Rad Antibodies, AHP799), 1:500; rabbit polyclonal anti-MLH1 (Santa Cruz Biotechnology, sc-11442), 1:100; mouse monoclonal anti-RNAPII (EMD Millipore, 05-952), 1:100; rabbit polyclonal anti-SLX4 (gift from Paula E. Cohen (Holloway et al., 2011)), 1:100; rabbit polyclonal anti-SYCP1 (Abcam, ab15090), 1:1500; mouse monoclonal anti-SYCP3 (Abcam, ab97642), 1:5,000; rabbit polyclonal anti-SYCP3 (Novus, NB300-231), 1:500; and rabbit polyclonal anti-TOPBP1 (gift from Junjie Chen (Yamane et al., 2002)), 1:500. After incubation of the primary antibodies, slides were washed three times for approximately five minutes each in PBST. Then, the slides were incubated with secondary antibodies conjugated to fluorophores (Thermo Fisher, Biotium, or Jackson ImmunoResearch). All secondary antibodies were diluted 1:500 in antibody dilution buffer. Slides were coated with 100 μL of antibody solution and then gently covered with Parafilm for approximately 30 minutes in humid chambers in darkness. Finally, slides were washed in PBST three times for five minutes each in darkness, then mounted in Vectashield (Vector Laboratories) containing 0.15% DAPI. Slides were either imaged immediately or stored at 4°C in darkness. For long-term storage, stained slides were kept at 4°C in darkness.

For double immunostaining using two primary antibodies from the same host species (rabbit polyclonal anti-FANCD2 antibody and rabbit polyclonal anti-RAD51 antibody), Fab fragments were used as suggested by the manufacturers of the secondary antibodies. Briefly, we performed immunostaining of anti-RAD51 antibody (six hours-to-overnight) and detected with Fab goat anti-rabbit IgG conjugated with Alexa Fluor 555 (Thermo Fisher). Then, slides were fixed in 1 mL of fresh, chilled 4% paraformaldehyde/1x PBS solution at room temperature for 10 minutes in a humid chamber. After briefly washing the fixed slides in PBST, we performed a second round of immunostaining with anti-FANCD2 antibody (six hours-to-overnight) followed by detection with Fab donkey anti-rabbit IgG conjugated with Alexa Fluor 647 (Thermo Fisher).

The specificity of FANCM antibodies was confirmed with peptide competition experiments using a FANCM peptide (CFDIQMLPNDLNQDRLKSDI) according to instructions at the Abcam website (http://www.abcam.com/protocols/blocking-with-immunizing-peptide-protocol-peptide-competition).

3D slide preparation and FISH

To conserve the morphology of meiotic chromatin, specialized slides that preserve the relative three-dimensional nuclear architecture of testicular germ cells were prepared as described (Namekawa, 2014; Namekawa and Lee, 2011; Namekawa et al., 2006). Briefly, seminiferous tubules underwent permeabilization, fixation, and then mechanical dissociation with fine-point tweezers before being cyto-spun onto positively-charged slides (e.g., Thermo Superfrost Plus, 12-550-15). Cot-1 RNA FISH was performed as described (Namekawa and Lee, 2011).

Microscopy and image analyses

All images of germ cells were acquired with an ECLIPSE Ti-E microscope (Nikon) and Zyla 5.5 sCMOS camera (Andor Technology), with 60x and 100x CFI Apochromat TIRF oil immersion lenses (Nikon), numerical aperture 1.40. Photoshop and Illustrator (CS6, Adobe) were used for composing figures. Primary spermatocytes were staged by staining for SYCP3 (described in detail in Fig. S1). For data analysis, the matched substage of meiotic prophase was analyzed in controls and mutants. All data were confirmed with at least three independent littermate pairs of mice.

Sample images of spermatocytes stained with anti-FANCD2, -BRCA2, -FANCM, and -MLH1 antibodies were blinded and manually scored with the ImageJ processing package Fiji (Schindelin et al., 2012). Sample images were blinded, scored, unblinded, and sorted through the following workflow: (1) Images were batch converted from the Nikon file format (.nd2) to the TIFF file format with ImageJ. (2) Composite TIFFs were batch blinded with ImageJ. (3) After focus counts were determined and recorded in Excel (Microsoft), the composite TIFF filenames were unblinded and sorted by genotypes and stages of meiotic prophase. (4) Data were imported to Prism 6 (GraphPad) for statistical analyses. Graphs of focus counts (FANCD2 and MLH1) were composed with Prism 6 and Illustrator; graphs of percentage accumulation (BRCA2 and FANCM) were composed with Excel and Illustrator.

H3K4me2, H3K9me2, and H3K9me3 signals were quantified with NIS-Elements Basic Research software (Nikon). Briefly, regions of interest (ROIs) were drawn around XY bodies, denoted as XY in Figs. 7 and S6, and prophase nuclei excluding the XY body, denoted as Au. (for "autosome region") in Figs. 7 and S6. The XY body and Au. ROIs were normalized to image background ROIs. For normalization of signals on a relative scale (0 to 1.5 for H3K4me2 and H3K9me2 analyses, 0 to 2 for H3K9me3 analyses), we calculated the mean of all diplotene XY body ROI signals and then divided all XY and Au. ROI signals by this value. This provided a relative value for ROI signals termed the "relative mean fluorescence intensity" (RMFI). The independent samples were combined and statistical analyses were run through Excel and Prism 6. RMFI graphs were composed with Prism 6 and Illustrator.

Cell culture
The human lymphoblast cell line PD20 (deficient for *FANCD2* (Timmers et al., 2001)) stably transduced with WT-FANCD2, the K561R mutant, or the empty pMMP retroviral vector (Li et al., 2010) were cultured in 10% fetal bovine serum RPMI 1640 medium containing 1 μg/mL puromycin. To induce monoubiquitination of FANCD2 in PD20 cells, DNA replication was arrested by treatment with 2 mM hydroxyurea, added from a 200 mM stock in growth medium kept at -20°C.

Western blotting
Whole testes and hydroxyurea-treated PD20 cells were dounce homogenized with RIPA buffer (10 mM Tris-HCl pH 7.5, 150 mM NaCl, 1 mM EDTA, 0.1% SDS, 0.1% NaDOC, 1% Triton X-100) containing protease inhibitor cOmplete (Roche). After the measurement of protein concentrations by the Bradford assay, lysates were mixed with an equal volume of 2x Laemmli sample buffer (prepared and stored at 4x concentration: 2% sodium dodecyl sulfate, 10% glycerol, 60 mM Tris-HCl pH 6.8, 1% v/v β-mercaptoethanol, and bromophenol blue), mixed with Benzonase (0.5 μL/100 μL lysate), and then incubated on ice for approximately 30 minutes, with gentle mixing every 10 minutes. If still viscous, the lysates were briefly sonicated on ice. Otherwise, the lysates were immediately boiled for 10 minutes. Using gels with 4% stacking and 6% running concentrations, SDS-PAGE was performed through 80 V in the stacking portion and 150 V in the running portion. Semi-dry transfer was performed with the Trans-Blot Turbo Transfer System (Bio-Rad, 1704155) by following the transfer method used by R&D Systems

(Bio-Techne; https://www.rndsystems.com/resources/protocols/western-blot-qc-protocol#Transfer). Using a PVDF membrane wetted in 100% methanol, semi-dry transfer was run for one hour with a constant current of 200 mA (15 V maximum). Afterwards, the membrane was blocked in StartingBlock Blocking Buffer (Thermo Scientific, 37538) for approximately 25 minutes, washed for 10 minutes in TBST two times, and then incubated with primary antibody. The following primary antibodies were diluted in TBST and then coated on the membrane for approximately one hour at room temperature: rabbit polyclonal anti-FANCD2 antibodies G33 (1:1000), E35 (1:1000), or Novus NB100-182 (1:1000). Then, the membrane was washed for 10 minutes in TBST three times, incubated in HRP-labeled anti-rabbit IgG diluted in TBST (1:5000), and incubated for one hour. After washing for 10 minutes in TBST four times, the membrane was visualized using Pierce ECL Western Blotting Substrate (Thermo Scientific, 32106) as instructed by the manufacturer.

References for this section

Broering, T.J., Alavattam, K.G., Sadreyev, R.I., Ichijima, Y., Kato, Y., Hasegawa, K., Camerini-Otero, R.D., Lee, J.T., Andreassen, P.R., and Namekawa, S.H. (2014). BRCA1 establishes DNA damage signaling and pericentric heterochromatin of the X chromosome in male meiosis. J Cell Biol *205*, 663-675.

Gallardo, T., Shirley, L., John, G.B., and Castrillon, D.H. (2007). Generation of a germ cell-specific mouse transgenic Cre line, Vasa-Cre. Genesis *45*, 413-417.

Holloway, J.K., Mohan, S., Balmus, G., Sun, X., Modzelewski, A., Borst, P.L., Freire, R., Weiss, R.S., and Cohen, P.E. (2011). Mammalian BTBD12 (SLX4) protects against genomic instability during mammalian spermatogenesis. PLoS Genet *7*, e1002094.

Ichijima, Y., Ichijima, M., Lou, Z., Nussenzweig, A., Camerini-Otero, R.D., Chen, J., Andreassen, P.R., and Namekawa, S.H. (2011). MDC1 directs chromosome-wide silencing of the sex chromosomes in male germ cells. Genes Dev *25*, 959-971.

Kato, Y., Alavattam, K.G., Sin, H.S., Meetei, A.R., Pang, Q., Andreassen, P.R., and Namekawa, S.H. (2015). FANCB is essential in the male germline and regulates H3K9 methylation on the sex chromosomes during meiosis. Human Molecular Genetics *24*, 5234-5249.!

Li, J., Du, W., Maynard, S., Andreassen, P.R., and Pang, Q. (2010). Oxidative stress-specific interaction between FANCD2 and FOXO3a. Blood *115*, 1545-1548.

Namekawa, S.H. (2014). Slide preparation method to preserve three-dimensional chromatin architecture of testicular germ cells. J Vis Exp, e50819.

Namekawa, S.H., and Lee, J.T. (2011). Detection of nascent RNA, single-copy DNA and protein localization by immunoFISH in mouse germ cells and preimplantation embryos. Nat Protoc *6*, 270-284.

Namekawa, S.H., Park, P.J., Zhang, L.F., Shima, J.E., McCarrey, J.R., Griswold, M.D., and Lee, J.T. (2006). Postmeiotic sex chromatin in the male germline of mice. Curr Biol *16*, 660-667.

Peters, A.H., Plug, A.W., van Vugt, M.J., and de Boer, P. (1997). A drying-down technique for the spreading of mammalian meiocytes from the male and female germline. Chromosome Res *5*, 66-68.

Schindelin, J., Arganda-Carreras, I., Frise, E., Kaynig, V., Longair, M., Pietzsch, T., Preibisch, S., Rueden, C., Saalfeld, S., Schmid, B*., et al.* (2012). Fiji: an open-source platform for biological-image analysis. Nature Methods *9*, 676-682.

Timmers, C., Taniguchi, T., Hejna, J., Reifsteck, C., Lucas, L., Bruun, D., Thayer, M., Cox, B., Olson, S., D'Andrea, A.D*., et al.* (2001). Positional cloning of a novel Fanconi anemia gene, FANCD2. Molecular Cell *7*, 241-248.

Xu, X., Wagner, K.U., Larson, D., Weaver, Z., Li, C., Ried, T., Hennighausen, L., Wynshaw-Boris, A., and Deng, C.X. (1999). Conditional mutation of Brca1 in mammary epithelial cells results in blunted ductal morphogenesis and tumour formation. Nat Genet *22*, 37-43.

Yamane, K., Wu, X., and Chen, J. (2002). A DNA damage-regulated BRCT-containing protein, TopBP1, is required for cell survival. Molecular and cellular biology *22*, 555-566.
