# Supplementary material for: Elucidation of the Fanconi Anemia Protein Network in Meiosis and Its Function in the Regulation of Histone Modifications
Source: Cell Rep. Author manuscript; Available in PMC 2016 Nov 4. (PMC5095620; doi:10.1016/j.celrep.2016.09.073)
Supplement: 2 [file NIHMS820305-supplement-2.pdf]

Cell Reports

Article

Elucidation of the Fanconi Anemia Protein Network in Meoisis and Its Function in the Regulation of Histone Modifications

Graphical Abstract

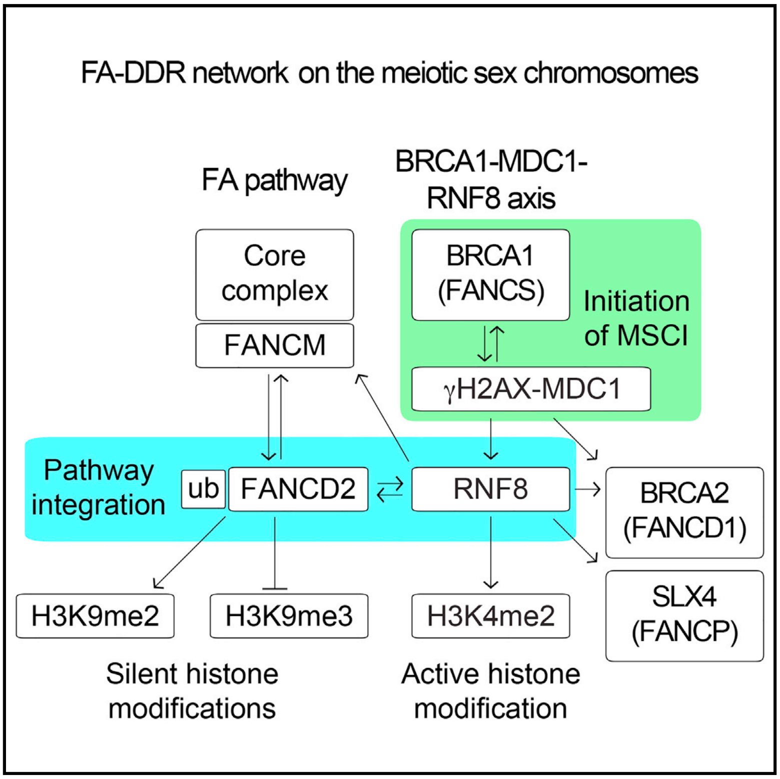

Highlights

- The FA-DDR network regulates the sex chromosomes during male meiosis
- FA proteins undergo both FA-core-complex-dependent and -independent regulation
- RNF8 is functionally linked with FANCD2 and integrates the FA-BRCA pathway
- FA core complex components and FANCD2 distinctly regulate H3K4 and H3K9 methylation

Authors

Kris G. Alavattam, Yasuko Kato, Ho-Su Sin, ..., Qishen Pang, Paul R. Andreassen, Satoshi H. Namekawa

Correspondence

satoshi.namekawa@cchmc.org

In Brief

Alavattam et al. reveal a regulatory network of Fanconi anemia (FA) and DNA damage response (DDR) proteins on the sex chromosomes during male meiosis. They show that FA proteins modulate distinct histone marks on the sex chromosomes and establish the sex chromosomes as a model to dissect the FA-DDR network.

CrossMark

Alavattam et al., 2016, Cell Reports *17*, 1141–1157
October 18, 2016 © 2016 The Author(s).
http://dx.doi.org/10.1016/j.celrep.2016.09.073

CellPress

OPEN ACCESS
CellPress

Elucidation of the Fanconi Anemia Protein Network in Meoisis and Its Function in the Regulation of Histone Modifications

Kris G. Alavattam,[1,3] Yasuko Kato,[1,3,4] Ho-Su Sin,[1,3,5] So Maezawa,[1,3] Ian J. Kowalski,[1,3,6] Fan Zhang,[2,3] Qishen Pang,[2,3] Paul R. Andreassen,[2,3] and Satoshi H. Namekawa[1,3,7,*]
[1]Division of Reproductive Sciences and Division of Developmental Biology, Perinatal Institute, Cincinnati, OH 45229, USA
[2]Division of Experimental Hematology and Cancer Biology, Cincinnati Children's Hospital Medical Center, Cincinnati, OH 45229, USA
[3]Department of Pediatrics, University of Cincinnati College of Medicine, Cincinnati, OH 49229, USA
[4]Present address: Department of Applied Biology, Institute for the Promotion of University Strategy, Kyoto Institute of Technology, Kyoto 606-8585, Japan
[5]Present address: Department of Developmental Biology and Department of Genetics, Stanford University School of Medicine, Stanford, CA 94305, USA
[6]Present address: University of Toledo College of Medicine, Toledo, OH 43614, USA
[7]Lead Contact
*Correspondence: satoshi.namekawa@cchmc.org
http://dx.doi.org/10.1016/j.celrep.2016.09.073

SUMMARY

Precise epigenetic regulation of the sex chromosomes is vital for the male germline. Here, we analyze meiosis in eight mouse models deficient for various DNA damage response (DDR) factors, including Fanconi anemia (FA) proteins. We reveal a network of FA and DDR proteins in which FA core factors FANCA, FANCB, and FANCC are essential for FANCD2 foci formation, whereas BRCA1 (FANCS), MDC1, and RNF8 are required for BRCA2 (FANCD1) and SLX4 (FANCP) accumulation on the sex chromosomes during meiosis. In addition, FA proteins modulate distinct histone marks on the sex chromosomes: FA core proteins and FANCD2 regulate H3K9 methylation, while FANCD2 and RNF8 function together to regulate H3K4 methylation independently of FA core proteins. Our data suggest that RNF8 integrates the FA-BRCA pathway. Taken together, our study reveals distinct functions for FA proteins and illuminates the male sex chromosomes as a model to dissect the function of the FA-BRCA pathway.

INTRODUCTION

In meiosis, homologous chromosomes undergo synapsis and recombination to promote genetic diversity in offspring. However, in male mammals, the sex chromosomes—X and Y—have vastly different morphologies and genetic content and are thus largely unsynapsed during meiosis. Instead, the sex chromosomes are transcriptionally silenced in a process known as meiotic sex chromosome inactivation (MSCI) (Ichijima et al., 2012; Turner, 2007). In MSCI, the sex chromosomes are compartmentalized together to form an XY body (also known as the sex body) and sequestered away from recombining autosomes. MSCI is initiated with the phosphorylation of histone variant H2AX at serine 139 (γH2AX) (Fernandez-Capetillo et al., 2003) and the near-simultaneous recruitment of binding partner MDC1 (Ichijima et al., 2011), a signaling mechanism that plays a crucial role in the DNA damage response (DDR) in somatic cells (Ciccia and Elledge, 2010; Polo and Jackson, 2011). Following the initiation of MSCI, extensive chromatin remodeling occurs on the sex chromosomes. This includes nucleosome replacement, such as H3.3 incorporation (van der Heijden et al., 2007), establishment of epigenetic modifications, and maintenance of chromosome-wide silencing through meiosis into post-meiotic stages (Greaves et al., 2006; Namekawa et al., 2006; Turner et al., 2006). Some DDR factors, such as BRCA1 and ATR, have been implicated in the initiation of MSCI (Broering et al., 2014; Fernandez-Capetillo et al., 2003; Ichijima et al., 2011; Royo et al., 2013; Turner et al., 2004). However, it remains unknown whether a DDR protein network functions in concert, as it does in the somatic DDR, to govern the sex chromosomes.

Fanconi anemia (FA) is a genetic disease associated with bone marrow failure, increased cancer susceptibility, and severe germline defects. Patients are said to have FA if they are deficient for any one of a growing number of FA proteins that function in a biochemical pathway known as the FA-BRCA pathway. This pathway is known to function in the resolution of a particularly harmful form of DNA damage, DNA interstrand crosslinks, in which the Watson and Crick strands become covalently linked (Kee and D'Andrea, 2010; Kottemann and Smogorzewska, 2013). There are currently 21 identified FA proteins (Bluteau et al., 2016; Kottemann and Smogorzewska, 2013; Park et al., 2016; Sawyer et al., 2015; Wang et al., 2015), comprising a network of proteins with distinct functions and properties. These include the FA core complex—FANCA, B, C, E, F, G, L, and M—which catalyzes the monoubiquitination of FANCD2 and FANCI in a biochemical pathway termed the FA pathway (Garcia-Higuera et al., 2001; Meetei et al., 2003; Sims et al.,

CrossMark

This is an open access article under the CC BY license (http://creativecommons.org/licenses/by/4.0/).

OPEN ACCESS
CellPress

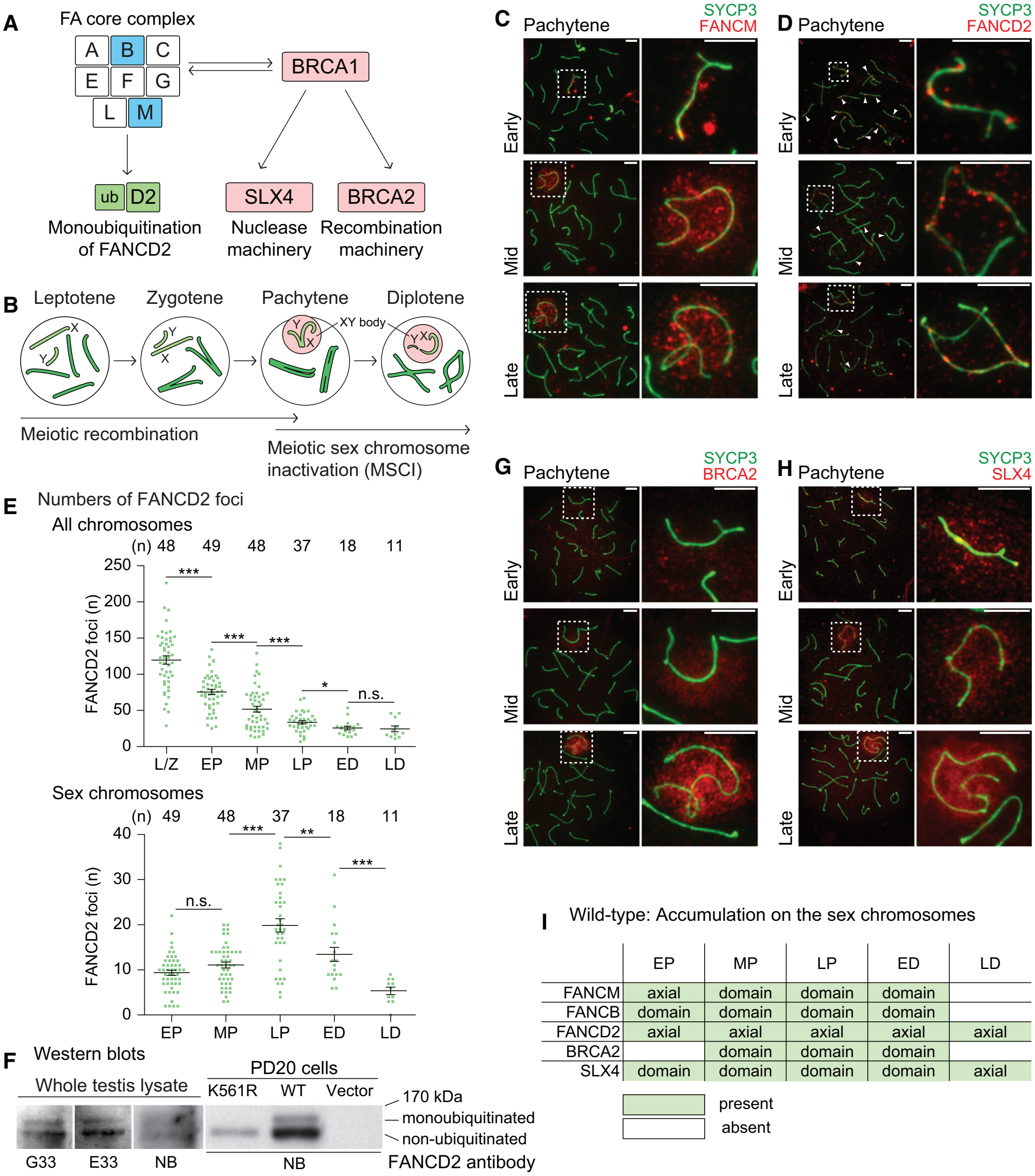

Figure 1. The FA-BRCA Pathway Is Activated on the Sex Chromosomes during Meiosis

(A) Schematic of the FA-BRCA pathway. FA proteins analyzed in this study are shown in color.

(B) Schematic of stages of meiotic prophase.

(C, D, G, and H) Immunostains using indicated antibodies in meiotic chromosome spreads from wild-type mice. SYCP3 is a marker for meiotic chromosome axes. Substages are labeled to the left. Dashed squares border sex chromosomes and are magnified to the right. Arrowheads: selected FANCD2 foci present on synapsed autosomes. Consistent results were obtained with n = 3 independent mice. Scale bars, 5 μm.

(E) Total number of FANCD2 foci on all chromosome axes (top) and on the sex chromosome axes (bottom) per spermatocyte for stages of meiotic prophase. Numbers of spermatocytes analyzed are noted above each graph. Bars represent means and SEMs. Data are aggregated from n = 6 wild-type adult mice. p values are derived from unpaired, two-tailed Student's t tests: n.s., not significant, $p > 0.05$; $^{*}p \leq 0.05$; $^{**}p \leq 0.01$; $^{***}p \leq 0.001$. Prophase spermatocyte stage abbreviations: L/Z, leptotene and zygotene; EP, early pachytene; MP, mid pachytene; LP, late pachytene; ED, early diplotene; LD, late diplotene.

(F) Western blot analysis with three independent anti-FANCD2 antibodies (G33, E33, and Novus NB100-182 antibody: NB). K561R, PD20 cells expressing a mutated form of FANCD2 incapable of monoubiquitination; WT, PD20 cells complemented with wild-type *FANCD2*; Vector, PD20 cells containing empty vector.

(legend continued on next page)

2007; Smogorzewska et al., 2007). Also included are breast cancer susceptibility proteins, such as BRCA1 (FANCS) and BRCA2 (FANCD1) (Howlett et al., 2002; Park et al., 2014b; Sawyer et al., 2015), and SLX4 (FANCP), a scaffold for endonucleases such as XPF (FANCQ) (Kim et al., 2011; Stoepker et al., 2011). It remains largely unknown how these diverse proteins relate to each other to function in the broader FA-BRCA pathway, and how proteins in this pathway relate to proteins in other DDR pathways. In this context, we recently demonstrated that a member of the FA core complex, FANCB, accumulates on the XY body and regulates H3K9 methylation (Kato et al., 2015). Because of the involvement of FANCB in the regulation of the sex chromosomes (Kato et al., 2015), we reasoned that the FA-BRCA pathway may regulate the meiotic sex chromosomes.

To test whether the FA-BRCA pathway regulates MSCI, we first determined that FA proteins accumulate on the sex chromosomes in coordinated temporal and spatial arrangements and demonstrated that the FA pathway is activated during meiosis as shown by FANCD2 monoubiquitination. To determine the functions of the broader FA-BRCA pathway in meiosis, and whether the different FA proteins are epistatic, we systematically analyzed eight mouse models deficient for various DDR factors, including several FA proteins. Our findings reveal that a network of FA and related DDR proteins, MDC1 and RNF8, functions in the epigenetic programming of the sex chromosomes. We term this network the FA-DDR network. Further, our work provides several mechanistic insights into how this network is regulated. Based upon our findings, the meiotic sex chromosomes have emerged as a model that can yield important insights into the functions of the FA-BRCA pathway, including its roles in the somatic DDR.

RESULTS

Fanconi Anemia Proteins Accumulate on the Sex Chromosomes in Coordinated Temporal and Spatial Arrangements

To determine the relationship of the FA pathway to the sex chromosomes during meiosis, we performed immunofluorescence microscopy of meiotic chromosome spreads, analyzing the localization of FA proteins. We judged each stage of meiotic prophase based on the precise appearance of chromosome axes (summarized in Figure S1A). We tested various antibodies against proteins that are components of the FA core complex (Figure 1A). At the onset of the pachytene stage, MSCI and meiotic recombination are distinctly regulated (Figure 1B). Among the FA core proteins, we found the partial accumulation of FANCM on the axes of the sex chromosomes (termed XY axes hereafter) during meiosis (Figure 1C). FANCM, a helicase that binds single-strand DNA, is thought to be recruited to replication forks stalled by DNA interstrand crosslinks in the somatic DDR (Bakker et al., 2009). The specificity of the anti-FANCM antibody (FARF D3823) was confirmed with a competition experiment using a FANCM peptide that matches the epitope region (Figure S1B). FANCM accumulation on the sex chromosome axes begins in the early pachytene stage and spreads onto the entire domain of the X and Y chromosomes (termed XY chromatin hereafter) through the early diplotene stage (Figures 1C and S1C). As spermatocytes progress through the remainder of prophase, FANCM is gradually lost from the XY chromatin (Figure S1C). In accord with this finding, our recent study demonstrated that FANCB, another FA core complex protein, accumulated on the sex chromosomes beginning in the early pachytene stage (Kato et al., 2015). Although we could not detect immunofluorescence signals for other core proteins during meiosis, our observations of FANCM and FANCB raised the possibility that the FA core complex is involved in the regulation of the sex chromosomes during meiosis.

Next, we examined whether the FA pathway is activated on the sex chromosomes. In somatic cells, activation of the FA pathway is measured by core complex-mediated monoubiquitination of FANCD2 (Figure 1A), which is followed by foci formation on chromatin (Garcia-Higuera et al., 2001; Taniguchi et al., 2002). Consistent with previous reports (An et al., 2012; Garcia-Higuera et al., 2001), FANCD2 foci localized on both autosome and sex chromosome axes during meiosis (Figure 1D). During the transition from the leptotene to zygotene stages, FANCD2 foci accumulated on the synapsed axes of autosomes and gradually decreased on autosomes through the remainder of meiotic prophase (Figures 1D, arrowheads, and 1E). On the other hand, we found that FANCD2 foci on the XY axes are regulated apart from those on autosomes. A small number of FANCD2 foci accumulated on the XY axes in the early pachytene stage and were amplified along the XY axes through the late pachytene stage before decreasing through the remainder of prophase (Figures 1D and 1E). Consistent with the appearance of FANCD2 foci, we detected monoubiquitinated FANCD2 by western blotting using the crude extract of wild-type mouse testes with three independent anti-FANCD2 antibodies (Figure 1F). We validated the presence of monoubiquitination by comparison with the somatic DDR. In brief, we blotted lysate from PD20 cells, a human lymphoblast cell line derived from an FA patient deficient for *FANCD2* (Timmers et al., 2001), that were reconstituted with different forms of FANCD2. Before producing the lysates, the PD20 variants were treated with hydroxyurea to induce stalled replication forks, leading to DNA damage and thus activation of the FA pathway. Monoubiquitination was observed in PD20 cells reconstituted with wild-type FANCD2 but not those that contained a non-ubiquitinable form of FANCD2, K561R (Figure 1F). Mono- and non-ubiquitinated FANCD2 bands from wild-type whole testis lysate co-migrated with those in reconstituted PD20 cells (Figure 1F). Although testes also contain cells that are not in meiosis, and since it has been demonstrated, using the K561R mutant, that monoubiquitination is required for

(I) Summary of temporal and spatial staining patterns for anti-FA protein antibodies on the sex chromosomes of wild-type mice. Axial, FA factors spread along XY axes. Domain, FA factors spread along XY axes and through XY chromatin. For comparison, FANCB results from our recent study (Kato et al., 2015) are summarized here.
See also Figure S1.

FANCD2 foci formation in somatic cells (Garcia-Higuera et al., 2001), we infer that FANCD2 is monoubiquitinated in meiosis. In support of our conclusion, a previous study also detected monoubiquitinated FANCD2 in testis lysate (Houghtaling et al., 2003). Together, these results suggest that the FA pathway is activated during normal meiosis and is distinctly regulated between autosomes and the sex chromosomes.

To further examine the involvement of FA proteins on sex chromosomes during meiosis, we reinvestigated the localization of two additional FA proteins reported to localize on the sex chromosomes during meiosis: BRCA2 (FANCD1) and SLX4 (FANCP) (Chen et al., 1998; Holloway et al., 2011). In the somatic DDR, BRCA2 is involved in the maintenance of genome stability via the homologous recombination pathway for double-strand DNA break repair (Moynahan et al., 2001). A previous study reported that BRCA2 is restricted to the XY axes during meiosis (Chen et al., 1998), but we found that BRCA2 localized to the XY chromatin in the late pachytene stage, after progressive accumulation on the X chromatin beginning in the mid pachytene stage (Figure 1G). Although we used a different anti-BRCA2 antibody than that reported by Chen et al., we confirmed the specificity of our anti-BRCA2 antibody based on immunoblots (Park et al., 2014a). It is interesting that FANCD2 and BRCA2 have distinct localization patterns on the meiotic sex chromosomes since these proteins strongly colocalize in somatic cells exposed to exogenous DNA damage (Wang et al., 2004). SLX4, a structure-specific endonuclease involved in the repair of various DNA lesions (Kottemann and Smogorzewska, 2013; Yamamoto et al., 2011), also localized on the XY chromatin in the late pachytene stage, consistent with a previous study (Holloway et al., 2011). SLX4 progressively increased in intensity as it spread through the XY chromatin during the pachytene stages (Figure 1H). The coordinated spatial and temporal localization of FANCM, FANCB, FANCD2, BRCA2, and SLX4 on the XY chromatin beginning in the early and mid pachytene stages (summarized in Figures 1I and S1C) suggests that the FA-BRCA pathway is activated on the sex chromosomes.

Fanconi Anemia Core Factors Are Essential for FANCD2 Foci on the Sex Chromosomes during Meiosis

Next, we sought to dissect the interrelationship of FA proteins on the sex chromosomes during meiosis. To determine whether members of the FA core complex (FANCA and FANCC) and FANCD2 are necessary for the recruitment of other FA proteins, we analyzed mutant mice deficient for FANCA (*Fanca*$^{-/-}$), FANCC (*Fancc*$^{-/-}$), and FANCD2 (*Fancd2*$^{-/-}$). The accumulation of FANCD2 foci was abolished on XY axes in *Fanca* and *Fancc* mutants (Figures 2A and S2A). Autosomal FANCD2 foci were also absent from *Fanca* and *Fancc* mutants (Figures 2A and S2A). This appears to parallel the role of the FA core complex in regulating FANCD2 recruitment to foci in somatic cells (Garcia-Higuera et al., 2001; Taniguchi et al., 2002). FANCD2 foci were absent from autosomal and XY axes in meiotic cells from *Fancd2* mutants (Figure 2B), demonstrating the specificity of the antibody used.

In contrast to FANCD2, the accumulation of BRCA2 on XY chromatin was unaffected in *Fanca*, *Fancc*, and *Fancd2* mutants (Figures 2C, 2D, and S2B). This is distinct from a previous report in which the assembly of BRCA2 foci into DNA damage foci induced by ionizing radiation depends on FANCD2 monoubiquitination in somatic cells (Wang et al., 2004). Further, SLX4 accumulation was unaffected in *Fanca*, *Fancc*, and *Fancd2* mutants (Figures 2E, 2F, and S2C). These data indicate that the accumulation of BRCA2 and SLX4 on sex chromosomes in meiosis is independent of the FA core complex and FANCD2 (Figures 2G and S2D). This is in contrast to a reported finding that the FA core complex and FANCD2 regulate SLX4 in the somatic DDR (Yamamoto et al., 2011). It should be noted that our recent study demonstrated that FANCB is essential for FANCD2 foci formation during meiosis but is dispensable for SLX4 localization on the sex chromosomes during meiosis (Kato et al., 2015). Therefore, the core factors FANCA, FANCB, and FANCC appear to have common functions on the sex chromosomes.

BRCA1 and MDC1 Are Required for the Accumulation of BRCA2 and SLX4 on the Sex Chromosomes during Meiosis

At the onset of MSCI, BRCA1 is a critical regulator of the DDR that recruits ATR (Turner et al., 2004) and establishes DDR signals along the unsynapsed axes (Broering et al., 2014). In somatic cells, BRCA1 is functionally linked to FA proteins (Folias et al., 2002; Garcia-Higuera et al., 2001; Zhang et al., 2010), and, indeed, *BRCA1* has been identified as an FA gene and designated *FANCS* (Sawyer et al., 2015). BRCA1 is required for the recruitment of FANCD2 to DNA interstrand crosslinks and other types of DNA damage in the somatic DDR (Garcia-Higuera et al., 2001; Vandenberg et al., 2003; Zhang et al., 2010). To investigate whether BRCA1 has a relevant function in regulating FA proteins in meiosis, we examined conditionally deleted mutants of *Brca1* established in our previous study (Broering et al., 2014). Since deletion of *Brca1* exon 11 has an embryonic lethal phenotype, *Brca1* exon 11 was conditionally deleted (*Brca1*cKO) using the germline-specific *Ddx4*-cre (also known as *Vasa*-cre) (Gallardo et al., 2007). FANCD2 foci were present, but not amplified, on the XY axes, and foci persisted on autosomal axes while their numbers decreased in control samples (Figures 3A and 3B). Thus, these data indicate a role for BRCA1 in the amplification of FANCD2 foci on the XY axes and the progressive resolution of FANCD2 foci from autosomes. On the other hand, the accumulation of BRCA2 on XY chromatin was abolished in the *Brca1*cKO (Figure 3C), consistent with the requirement of BRCA1 for BRCA2 foci formation in the somatic DDR (Chen et al., 1998; Zhang et al., 2009a, 2009b). Furthermore, in the *Brca1*cKO, accumulation of SLX4 on XY chromatin was abolished (Figure 3D), indicating that BRCA1 is required for SLX4 recruitment to XY chromatin. Although it has been reported that BRCA1 is not necessary for the recruitment of SLX4 to interstrand crosslinks in somatic cells (Lachaud et al., 2014), these data indicate that BRCA1 regulates SLX4 on the sex chromosomes. *Brca1*cKO spermatocytes undergo meiotic arrest at the mid pachytene stage and are eliminated soon afterward (Broering et al., 2014; Xu et al., 2003), but we conclude that the abrogation of BRCA2 and SLX4 localization is not due to meiotic arrest since we observed the beginnings of BRCA2 and SLX4 accumulation in, respectively, the mid and early pachytene stages of wild-type spermatocytes (Figures 1G, 1H, and S1C).

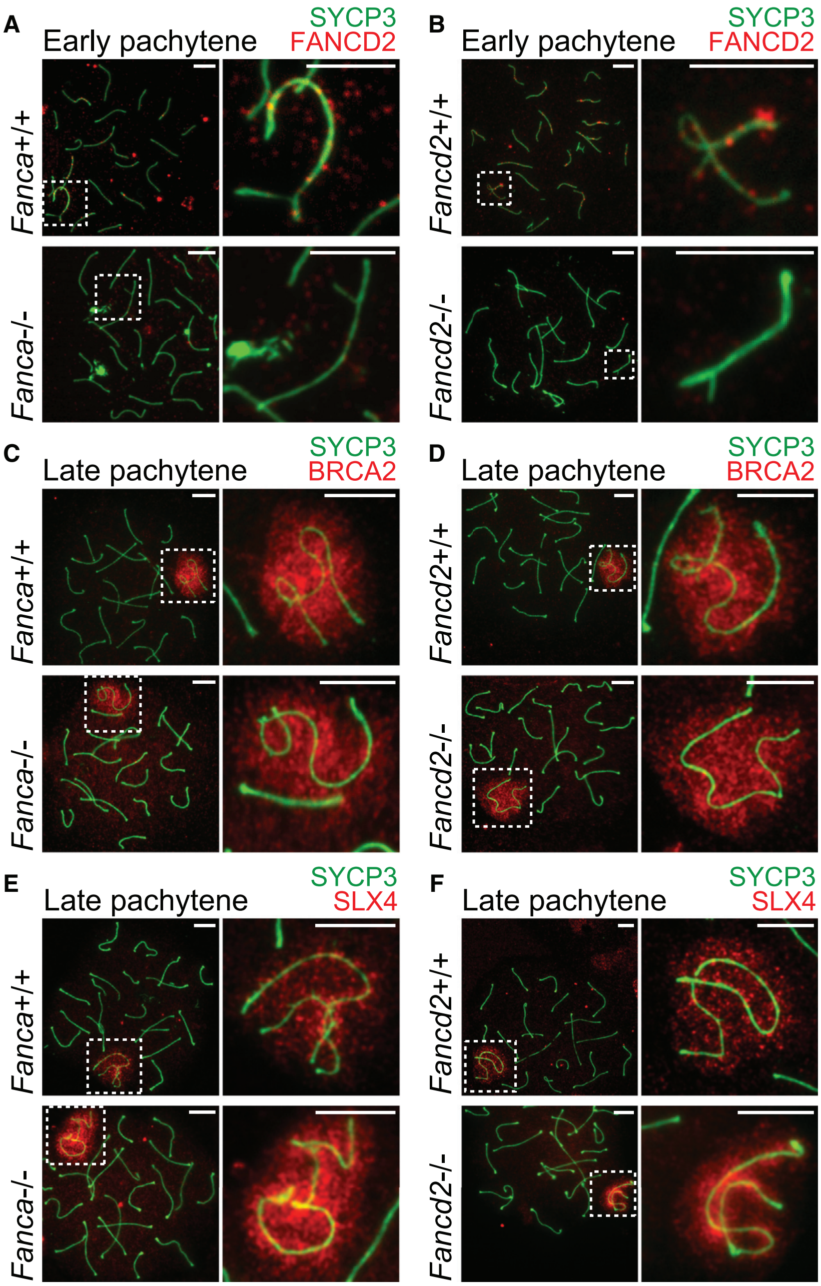

G *Fanca*-/-, *Fancd2*-/-: Accumulation on the sex chromosomes

	EP	MP	LP	ED	LD
FANCD2					
BRCA2		domain	domain	domain	
SLX4	domain	domain	domain	domain	axial

(shaded) present
(unshaded) absent

Figure 2. FA Core-Dependent and -Independent Functions of the FA Pathway

(A–F) Immunostains using indicated antibodies in meiotic chromosome spreads from *Fanca*$^{-/-}$ mice, *Fancd2*$^{-/-}$ mice, and wild-type littermate controls. Stages are labeled above, genotypes are labeled to the left. Dashed squares border sex chromosomes and are magnified to the right. Consistent results were obtained with n = 3 independent littermate pairs for each mouse model. Scale bars, 5 μm.

(G) Summary of temporal and spatial localization of anti-FA protein antibodies on the sex chromosomes in *Fanca*$^{-/-}$ and *Fancd2*$^{-/-}$ mice; summaries of localization in wild-type mice are shown in Figures 1I and S1C.

See also Figure S2.

OPEN
ACCESS
CellPress

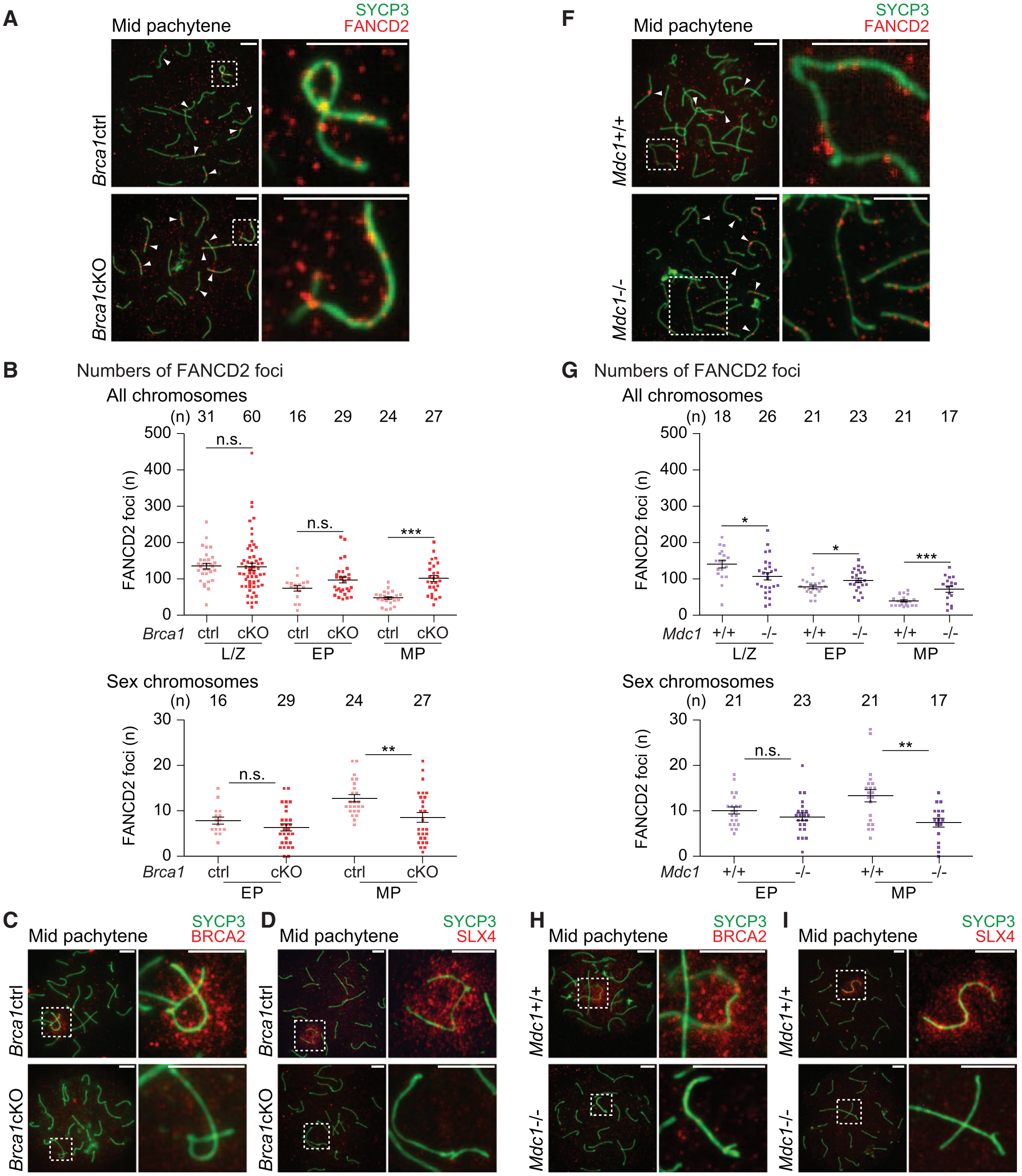

E *Brca1*cKO: Accumulation on the sex chromosomes

	EP	MP	
FANCD2	axial	axial	meiotic arrest
BRCA2			
SLX4			

present (green) — reduced (yellow) — absent (white)

J *Mdc1-/-*: Accumulation on the sex chromosomes

	EP	MP	
FANCD2	axial	axial	meiotic arrest
BRCA2			
SLX4			

present (green) — reduced (yellow) — absent (white)

Figure 3. BRCA1 and MDC1 Regulate the Localization of FA Proteins in Meiosis

(A, C, D, F, H, and I) Immunostains using indicated antibodies in meiotic chromosome spreads from *Brca1*cKO mice, *Mdc1*$^{-/-}$ mice, and wild-type or heterozygous littermate controls. Stages are labeled above; genotypes are labeled to the left. Dashed squares border sex chromosomes and are magnified to the right. Arrowheads: selected FANCD2 foci present on synapsed autosomes. Consistent results were obtained with n = 3 independent littermate pairs for each mouse model. Scale bars, 5 μm.

(legend continued on next page)

Thus, BRCA1 regulates amplification of FANCD2 foci on the XY axes and is required for the accumulation of BRCA2 and SLX4 on the XY chromatin (Figure 3E). Therefore, while FANCD2, BRCA2, and SLX4 are each regulated by BRCA1, examination of meiotic cells shows that BRCA1 has distinct roles in the recruitment of FANCD2 versus BRCA2 and SLX4. In particular, BRCA1 may be more important for the recruitment of BRCA2 and SLX4 than for the recruitment of FANCD2.

After the establishment of DDR signaling on the unsynapsed XY axes by BRCA1 (Broering et al., 2014), MDC1 is essential for the initiation of MSCI (Ichijima et al., 2011). MDC1 plays a crucial role in the somatic DDR (Goldberg et al., 2003; Lou et al., 2003; Stewart et al., 2003) and works in a feedback loop with the TOPBP1-ATR network to spread γH2AX to the chromosome-wide XY chromatin in the early pachytene stage (Ichijima et al., 2011). Because MDC1 recognizes XY chromatin at the onset of MSCI, we sought to determine whether MDC1 is required for the recruitment of FA proteins. Similar to *Brca1*cKO cells, FANCD2 foci were present in spermatocytes deficient for MDC1 (*Mdc1*$^{-/-}$), unamplified on the XY axes, and persistent on autosomal axes as spermatocytes progressed into the mid pachytene stage (Figures 3F and 3G). Thus, MDC1, like BRCA1, regulates amplification of FANCD2 foci on XY axes and the resolution of FANCD2 foci from autosomal axes. Additionally, the accumulation of both BRCA2 and SLX4 on XY chromatin was abolished in *Mdc1*$^{-/-}$ cells at meiosis (Figures 3H and 3I). Because *Mdc1*$^{-/-}$ spermatocytes undergo meiotic arrest at the mid pachytene stage, we conclude that the abolishment of BRCA2 and SLX4 accumulation is not due to meiotic arrest given that we did not observe the accumulation of BRCA2 and SLX4 in, respectively, the mid and early pachytene stages. And because BRCA1 localization was not disturbed in *Mdc1*$^{-/-}$ spermatocytes (Ichijima et al., 2011), BRCA1 is upstream of MDC1 in meiosis. Taken together, BRCA1 and MDC1 cooperate in the same pathway in the regulation of FANCD2 foci on chromosomes axes, as well as in the accumulation of BRCA2 and SLX4 on XY chromatin (Figure 3J). Because BRCA2 and SLX4 are not regulated by the FA core complex and FANCD2 (Figures 2 and S2), these results demonstrate that the functions of BRCA1 and MDC1 are uncoupled from that of the FA core complex and FANCD2.

RNF8 Regulates the Maintenance of FANCD2 and BRCA2 and Is Required for SLX4 Accumulation

On the sex chromosomes, the E3 ubiquitin ligase RNF8 works downstream of MDC1 and is required for ubiquitination of the XY chromatin. RNF8 is also required for subsequent active epigenetic modifications on the XY chromatin during meiosis and for gene activation in postmeiotic round spermatids (Sin et al., 2012). However, in the somatic DDR, MDC1 recruits and interacts with RNF8 to facilitate the recruitment of various downstream DDR factors (Huen et al., 2007; Kolas et al., 2007; Mailand et al., 2007; Zhang et al., 2012). Although RNF8 partially regulates the FA-BRCA pathway in the context of interstrand crosslink repair (Bick et al., 2016; Yan et al., 2012; Zhang et al., 2012), it remains unknown whether RNF8 regulates FA proteins during meiosis. To investigate this possibility, we analyzed the recruitment of FA proteins in male mutant mice deficient for RNF8 (*Rnf8*$^{-/-}$). The accumulation of FANCD2 foci on XY axes was undisturbed in the early pachytene stage of *Rnf8*$^{-/-}$ spermatocytes (Figures 4A and 4B). A reduced number of foci appeared on chromosome axes in the midst of condensation and synapsis in the leptotene/zygotene stages (Figure 4B). Strikingly, as *Rnf8*$^{-/-}$ spermatocytes progressed through the three pachytene stages, FANCD2 foci were not amplified along the XY axes through the late pachytene stage (Figures 4A and 4B)—in stark contrast to wild-type late pachytene spermatocytes (Figures 1D and 1E). Thus, these data suggest that RNF8 is required for the amplification of FANCD2 foci on the XY axes. This is in contrast to the absence of FANCD2 foci on the XY axes in *Fanca* and *Fancc* mutants (Figures 2A and S2A).

Interestingly, we also observed severe impairment of BRCA2 accumulation in *Rnf8*$^{-/-}$ spermatocytes. In contrast to wild-type spermatocytes (Figures 1G and S1C), the initial accumulation of BRCA2 was abrogated in most *Rnf8*$^{-/-}$ samples (Figures 4C and 4D). However, as the late pachytene stage transitioned into the early diplotene stage, BRCA2 accumulated and spread over portions of the XY chromatin in *Rnf8*$^{-/-}$ spermatocytes with decreased efficiency compared to wild-type controls (Figures 4C and 4D). SLX4 was more severely affected in *Rnf8*$^{-/-}$ cells: it did not accumulate on XY chromatin at all (Figure 4E). Thus, in addition to the RNF8-dependent amplification of FANCD2 foci on XY axes, RNF8 modulates the accumulation and maintenance of BRCA2 and is essential for the accumulation of SLX4 on the XY chromatin (Figure 4F). Because RNF8 works downstream of BRCA1 and MDC1 on the sex chromosomes (Lu et al., 2013; Sin et al., 2012), these results suggest that RNF8 is a key DDR factor regulating FANCD2. Further, our data indicate a two-step mechanism for the formation of FANCD2 foci (Figure 4G): the first step is FA core dependent and regulates the initial accumulation of FANCD2 foci beginning in the leptotene stage, and the second step regulates the amplification of FANCD2 foci on the XY axes through the BRCA1-MDC1-RNF8 signaling axis.

The Initial Accumulation of FANCD2 Foci on XY Axes Likely Represents Persistent DNA Double-Strand Breaks

We next examined whether the initial accumulation of FANCD2 foci on XY axes represents persistent DNA double-strand breaks (DSBs), which may serve as landmarks to target meiotic

(B and G) Total number of FANCD2 foci on all chromosome axes (top) and on the sex chromosome axes (bottom) per spermatocyte for stages of meiotic prophase. Numbers of spermatocytes analyzed are noted above each graph. Bars represent means and SEMs. Data are aggregated from n = 4 littermate pairs of *Brca1* mice, n = 3 littermate pairs of *Mdc1* mice. p values are derived from unpaired, two-tailed Student's t tests: n.s., not significant, $p > 0.05$; $^{*}p \leq 0.05$; $^{**}p \leq 0.01$; $^{***}p \leq 0.001$. L/Z, leptotene and zygotene; EP, early pachytene; MP, mid pachytene.

(E and J) Summaries of temporal and spatial localization of anti-FA protein antibodies on the sex chromosomes in *Brca1*cKO and *Mdc1*$^{-/-}$ mice; summaries of localization in wild-type mice are shown in Figures 1I and S1C. Spermatocytes from the *Brca1*cKO and *Mdc1*$^{-/-}$ models undergo meiotic arrest and apoptosis after the mid pachytene stage, designated by "meiotic arrest."

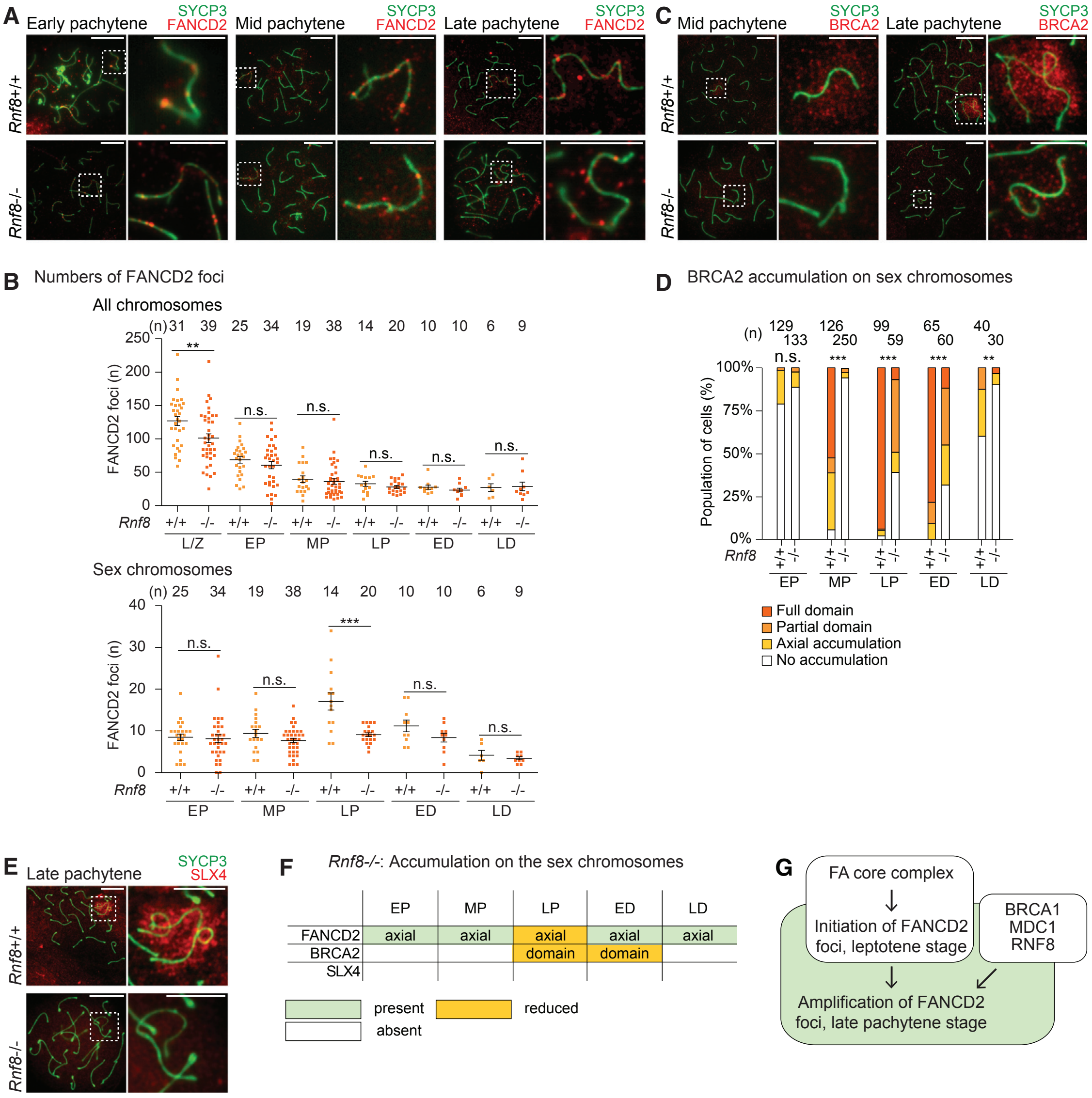

Figure 4. RNF8 Regulates the FA-BRCA Pathway

(A, C, and E) Immunostains using indicated antibodies in meiotic chromosome spreads from *Rnf8*$^{-/-}$ mice and wild-type littermate controls. Stages are labeled above, genotypes are labeled to the left. Dashed squares border sex chromosomes and are magnified to the right. Consistent results were obtained with n = 9 independent littermate pairs. Scale bars, 5 μm.

(B) Total number of FANCD2 foci on all chromosome axes (top) and on the sex chromosome axes (bottom) per spermatocyte for stages of meiotic prophase. Numbers of spermatocytes analyzed are noted above each graph. Bars represent means and SEMs. Data are aggregated from n = 4 littermate pairs. p values are derived from unpaired, two-tailed Student's t tests: n.s., not significant, $p > 0.05$; *$p \leq 0.05$; **$p \leq 0.01$; ***$p \leq 0.001$. L/Z, leptotene and zygotene; EP, early pachytene; MP, mid pachytene; LP, late pachytene; ED, early diplotene; LD, late diplotene.

(D) Categorical staining patterns for BRCA2 accumulation on sex chromosomes in pachytene and diplotene spermatocytes. Numbers of spermatocytes analyzed are noted above each graph. Accumulation patterns: Full domain, covers entirety of XY axes and chromatin; Partial domain, covers XY axes and portions of XY chromatin; Axial accumulation, covers XY axes; No accumulation, depletion from XY axes and chromatin. Data are aggregated from n = 6 littermate pairs. p values are derived from Pearson's chi-square test: n.s., not significant, $p > 0.05$; *$p \leq 0.05$; **$p \leq 0.01$; ***$p \leq 0.001$.

(F) Summary of temporal and spatial localization of anti-FA protein antibodies on the sex chromosomes in *Rnf8*$^{-/-}$ mice; summaries of localization in wild-type mice are shown in Figures 1I and S1C.

(G) Model of two-step amplification of FANCD2 foci on the sex chromosomes.

silencing to the sex chromosomes (Broering et al., 2014; Carofiglio et al., 2013; Inagaki et al., 2010). Given the possible recruitment of RAD51 to sites of unrepaired DSBs on the XY axes (Inagaki et al., 2010), we examined whether the initial accumulation of FANCD2 foci overlapped with that of RAD51, which repairs DSBs by homologous recombination. In normal early pachytene spermatocytes, the initial accumulation of FANCD2 foci on XY axes largely overlapped with that of RAD51 foci

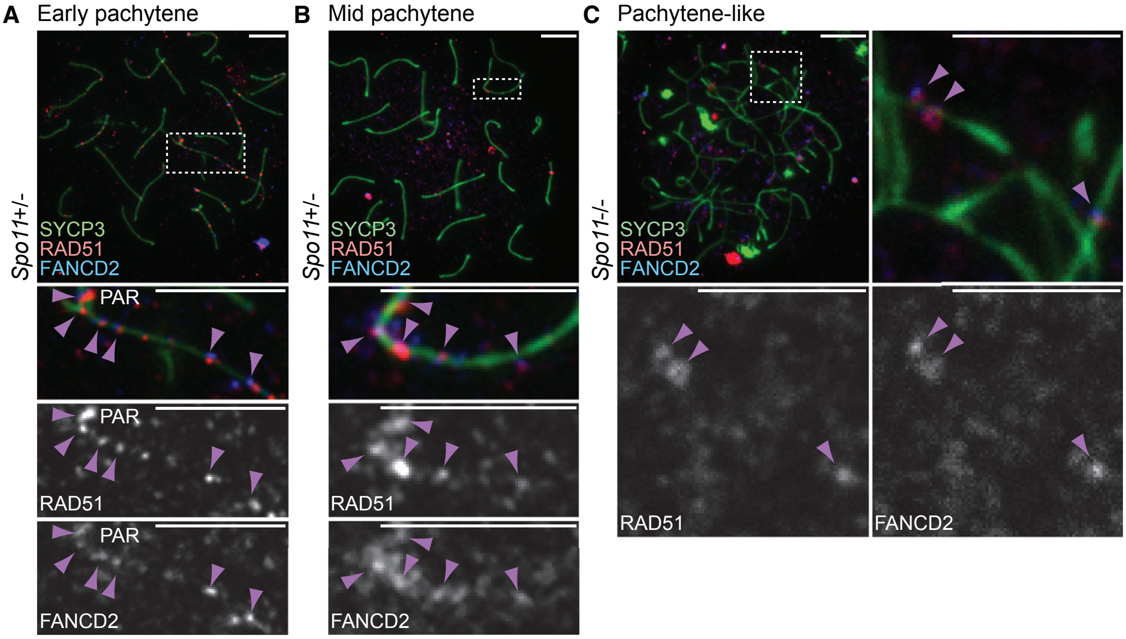

Figure 5. FANCD2 Colocalizes with RAD51 at Sites of Persistent DSBs

(A–C) Immunostains using indicated antibodies in meiotic chromosome spreads from *Spo11*$^{-/-}$ mice and control littermates. Stages of meiotic prophase are labeled above images; genotypes are labeled to the left of images. Dashed boxes border selected nuclear regions and are magnified below. Arrowheads: colocalization of FANCD2 and RAD51. Consistent results were obtained with n = 3 independent littermate pairs. PAR, pseudo-autosomal region. Scale bars, 5 μm. See also Figure S3.

(Figure 5A), suggesting that the initial FANCD2 foci on XY axes represent sites of unrepaired DSBs. FANCD2 colocalizes with RAD51 in spermatocytes transitioning from the mid to late pachytene stages as well (Figure 5B).

To further elaborate on the conclusion that FANCD2 foci occupy sites of unrepaired DSBs on XY axes, we performed immunofluorescence colocalization experiments using spermatocytes from the *Spo11* knockout model (*Spo11*$^{-/-}$), which is defective for SPO11-dependent DSBs (Baudat et al., 2000; Romanienko and Camerini-Otero, 2000). SPO11 is responsible for generating programmed DSBs for meiotic recombination and is thus required for proper chromosome synapsis. Interestingly, a small number of RAD51 foci were reported in *Spo11*$^{-/-}$ spermatocytes, and these presumably represent SPO11-independent DNA repair foci (Carofiglio et al., 2013). We found that a reduced number of FANCD2 foci tend to colocalize with RAD51 foci in *Spo11*$^{-/-}$ spermatocytes (Figure 5C), suggesting that FANCD2 accumulates at SPO11-independent DNA repair foci.

SPO11-independent DNA repair foci were proposed to be the cause of the ectopic meiotic silencing that occurs in *Spo11*$^{-/-}$ spermatocytes (Carofiglio et al., 2013). Sites of ectopic meiotic silencing are referred to as pseudo sex bodies since they are known to cover autosome chromatin (Barchi et al., 2005; Bellani et al., 2005). In support of this notion, we found that the majority of observed FANCD2 foci colocalized with MDC1 domains: XY chromatin in wild-type spermatocytes (Figure S3A) and pseudo sex bodies in *Spo11*$^{-/-}$ spermatocytes (Figure S3B). Together, these data support the possibility that the initial accumulation of FANCD2 foci represents persistent DSBs, which function to target the silencing machinery to unsynapsed chromatin, including the sex chromosomes, in meiotic prophase.

FANCD2 Cooperates with the BRCA1-MDC1-RNF8 Axis for the Accumulation of FANCM

In the course of our analyses of the sex chromosomes, we observed a dynamic temporal and spatial accumulation pattern for FANCM (Figures 1C and S1C), an FA protein associated with the FA core complex. To further define the epistatic relationship of the FA proteins, we designed experiments to determine whether proteins in a broad FA-DDR network—composed of the FA core complex, FANCD2, and the BRCA1-MDC1-RNF8 signaling axis—regulate FANCM. We scored the accumulation patterns of FANCM in spermatocytes at different time points of meiotic prophase and ran Pearson's chi-square test to identify categorical differences in accumulation between control and mutant samples. While FANCA, FANCB, and FANCC are dispensable for FANCM accumulation and maintenance (Figures S4A–S4D; data not shown), FANCD2 is necessary for the proper accumulation and maintenance of FANCM signals on the sex chromosomes (Figures 6A and 6B). Beginning in the early pachytene stage, FANCM accumulates on the sex chromosomes of *Fancd2*$^{-/-}$ spermatocytes with reduced efficiency (Figure 6B). As prophase progresses, FANCM fails to spread through the XY chromatin domain and, instead, is progressively lost from the XY chromatin and axes (Figure 6B). Given the normal accumulation and spreading of FANCM in the *Fanca*, *Fancb*, and *Fancc* knockout models (Figures S4A–S4D; data not shown), these data indicate a function for FANCD2 that is independent

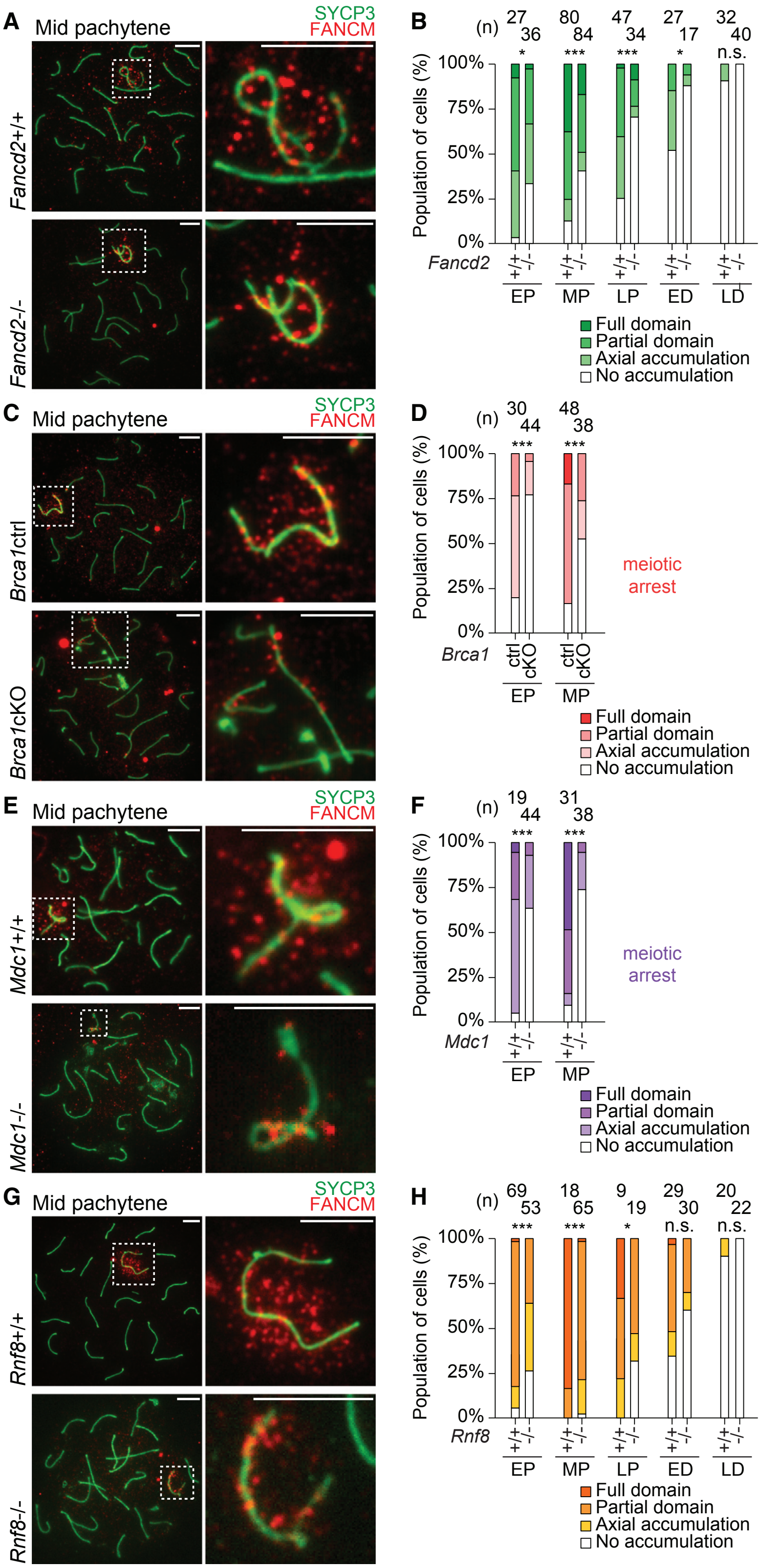

Figure 6. FANCD2 Cooperates with the BRCA1-MDC1-RNF8 Axis to Regulate FANCM on the Sex Chromosomes

(A, C, E, and G) Immunostains using indicated antibodies in meiotic chromosome from *Fancd2*$^{-/-}$ (A), *Brca1*cKO (C), *Mdc1*$^{-/-}$ (E), and *Rnf8*$^{-/-}$ mice (G), and corresponding wild-type littermate controls. Stages are labeled above; genotypes are labeled to the left. Dashed squares border sex chromosomes and are magnified to the right. Consistent results were obtained with n = 5 *Fancd2*, n = 4 *Brca1*, n = 4 *Mdc1*, and n = 4 *Rnf8* littermate pairs. Scale bars, 5 μm.

(B, D, F, and H) Categorical staining patterns for FANCM accumulation on sex chromosomes of *Fancd2* (B), *Brca1* (D), *Mdc1* (F), and *Rnf8* (H) spermatocytes. Numbers of spermatocytes analyzed are noted above each graph. Accumulation scored according to criteria described in the legend for Figure 4D. Data are aggregated from n = 5 *Fancd2*, n = 4 *Brca1*, n = 4 *Mdc1*, and n = 4 *Rnf8* littermate pairs. p values are derived from Pearson's chi-square tests: n.s., not significant, $p > 0.05$; $*p \leq 0.05$; $**p \leq 0.01$; $***p \leq 0.001$.

See also Figures S4 and S5.

of the FA core complex. Strikingly, we observed a more severe phenotype in the *Brca1*cKO and *Mdc1*$^{-/-}$ models: our analyses revealed a drastic reduction of FANCM accumulation and spreading over the XY chromatin when compared to controls (Figures 6C–6F). Our analyses also implicated RNF8 in the accumulation and maintenance of FANCM on the sex chromosomes: although the phenotype was not as severe as those of *Brca1*cKO and *Mdc1*$^{-/-}$ spermatocytes, FANCM accumulation and maintenance was disrupted in *Rnf8*$^{-/-}$ samples (Figures 6G and 6H). The extent of this disruption was similar to that observed in *Fancd2*$^{-/-}$ spermatocytes. Together, these data suggest that FANCD2, independent of the FA core complex, cooperates with the BRCA1-MDC1-RNF8 axis to regulate the accumulation and maintenance of FANCM on the sex chromosomes.

The FA Core Complex and FANCD2 Are Nonessential for Upstream DDR Events in MSCI and Meiotic Recombination

Given the possibility that FANCD2 functions independently of the FA core complex on the sex chromosomes, we next examined whether the core complex-FANCD2 axis, also known as the FA pathway, regulates early DDR events that occur on the sex chromosomes during MSCI. For these experiments, we used the *Fancd2*$^{-/-}$ model as a proxy for loss of function of the FA pathway. We found that FANCD2 does not regulate the accumulation of early DDR factors that are crucial for the initiation of MSCI, including BRCA1, ATR, TOPBP1, γH2AX, and MDC1 (Figures S5A–S5E). In our previous publication (Kato et al., 2015), we established that the FA core protein FANCB is also dispensable for the accumulation of these DDR factors. Consistent with our FANCB report, upstream DDR events occurred normally in *Fanca*$^{-/-}$ and *Fancc*$^{-/-}$ spermatocytes (data not shown). Together, these results indicate that the FA core complex and FANCD2 are dispensable for upstream DDR events on the sex chromosomes. Furthermore, these results explain why meiotic arrest is not induced in FA mutant mice: we infer that the FA pathway works downstream of γH2AX signaling and the initiation of MSCI, so FA deficiencies bypass meiotic arrest.

Because of the accumulation of FANCD2 foci on autosomes, we next determined whether the FA pathway is involved in the resolution of DSBs in meiotic recombination. We examined two factors involved in DSB resolution: RAD51, an upstream recombinase in the DSB repair pathway, and MLH1, a downstream mismatch repair protein that catalyzes crossover recombination. We found the unperturbed formation of RAD51 and MLH1 foci in *Fancd2*$^{-/-}$ and control spermatocytes (Figures S5F–S5H). Consistent with the normal formation of RAD51 and MLH1 foci, we detected normal chromosome synapsis in *Fancd2*$^{-/-}$ spermatocytes, as detected by double immunostaining for SYCP3 and SYCP1, a factor present at synapsed meiotic axes (Figure S5I). Combined, these data indicate a nonessential role for the FA pathway in meiotic recombination and chromosome synapsis.

FA Core-Dependent Regulation of H3K9 Methylation and FA Core-Independent Regulation of H3K4 Methylation

Because the FA pathway is not required for upstream DDR events, we next investigated possible downstream steps. Following the accumulation of DDR factors, epigenetic modifiers are recruited and histone modifications that can regulate transcription are established on the XY chromatin during meiosis (Ichijima et al., 2012). Our previous studies demonstrated that DDR factors regulate histone modifications on the XY chromatin: FANCB regulates H3K9 methylation, and RNF8 regulates active epigenetic modifications (Kato et al., 2015; Sin et al., 2012). To determine whether there is a general role for the FA pathway in epigenetic programming, we tested the localization of several histone modifications on XY chromatin by immunostaining *Fanca*$^{-/-}$, *Fancc*$^{-/-}$, and *Fancd2*$^{-/-}$ spermatocytes. Because of the relationship between the FA proteins and RNF8, we first investigated the possibility that the FA pathway is functionally linked with RNF8 by testing the RNF8-dependent active epigenetic modification H3K4me2 (Sin et al., 2012). In *Fanca*$^{-/-}$ and *Fancc*$^{-/-}$ spermatocytes, H3K4me2 accumulation on XY chromatin during the pachytene-to-diplotene transition was not affected (Figures 7A, 7B, S6A, and S6B). However, in *Fancd2*$^{-/-}$ cells, accumulation of H3K4me2 was decreased on XY chromatin during the pachytene-to-diplotene transition (Figures 7C and 7D). These results were confirmed through the quantification of relative mean fluorescence intensity (RMFI) from $n \geq 3$ sets of independent samples for *Fanca*, *Fancc*, and *Fancd2* mutants and littermate controls. Thus, FANCD2 regulates H3K4me2 accumulation on the XY chromatin, whereas the FA core factors do not. Since RNF8 is involved in the amplification of FANCD2 on sex chromosomes (Figures 4A and 4B), FANCD2 may act downstream to mediate RNF8-dependent H3K4me2 accumulation. Together, these results suggest that RNF8 is a central factor that integrates the broader FA-BRCA pathway.

Next, we examined whether the FA pathway regulates the silent epigenetic modification H3K9me2, which accumulates on the XY chromatin in the transition from the pachytene to diplotene stages (Khalil et al., 2004; Namekawa et al., 2006). In *Fanca*$^{-/-}$, *Fancc*$^{-/-}$, and *Fancd2*$^{-/-}$ spermatocytes, H3K9me2 was diminished on the sex chromosomes during this transition in comparison to wild-type sex chromosomes (Figures 7E–7H, S6C, and S6D). Together with our recent study demonstrating that FANCB regulates H3K9me2 on XY chromatin (Kato et al., 2015), these results suggest that the FA core complex and FANCD2 are required for the regulation of H3K9me2 on XY chromatin during the pachytene-to-diplotene transition.

Also, because we recently found that FANCB negatively regulates H3K9me3 (Kato et al., 2015), we investigated whether the FA pathway regulates H3K9me3. In wild-type spermatocytes, H3K9me3 initially accumulates on XY chromatin in the early pachytene stage and disappears in the mid pachytene stage due to histone H3 replacement (van der Heijden et al., 2007); then, in the late diplotene stage, H3K9me3 reaccumulates on the XY chromatin (van der Heijden et al., 2007). In *Fanca*$^{-/-}$, *Fancc*$^{-/-}$, and *Fancd2*$^{-/-}$ spermatocytes, H3K9me3 intensity was increased on both early pachytene and late diplotene sex chromosomes in comparison to wild-type sex chromosomes (Figures 7I–7L, S6E, and S6F). These results suggest that the FA pathway negatively regulates H3K9me3 both at the early pachytene and late diplotene stages. Taken together, we conclude that the FA pathway positively regulates H3K9me2 and negatively regulates H3K9me3 on XY chromatin. Therefore,

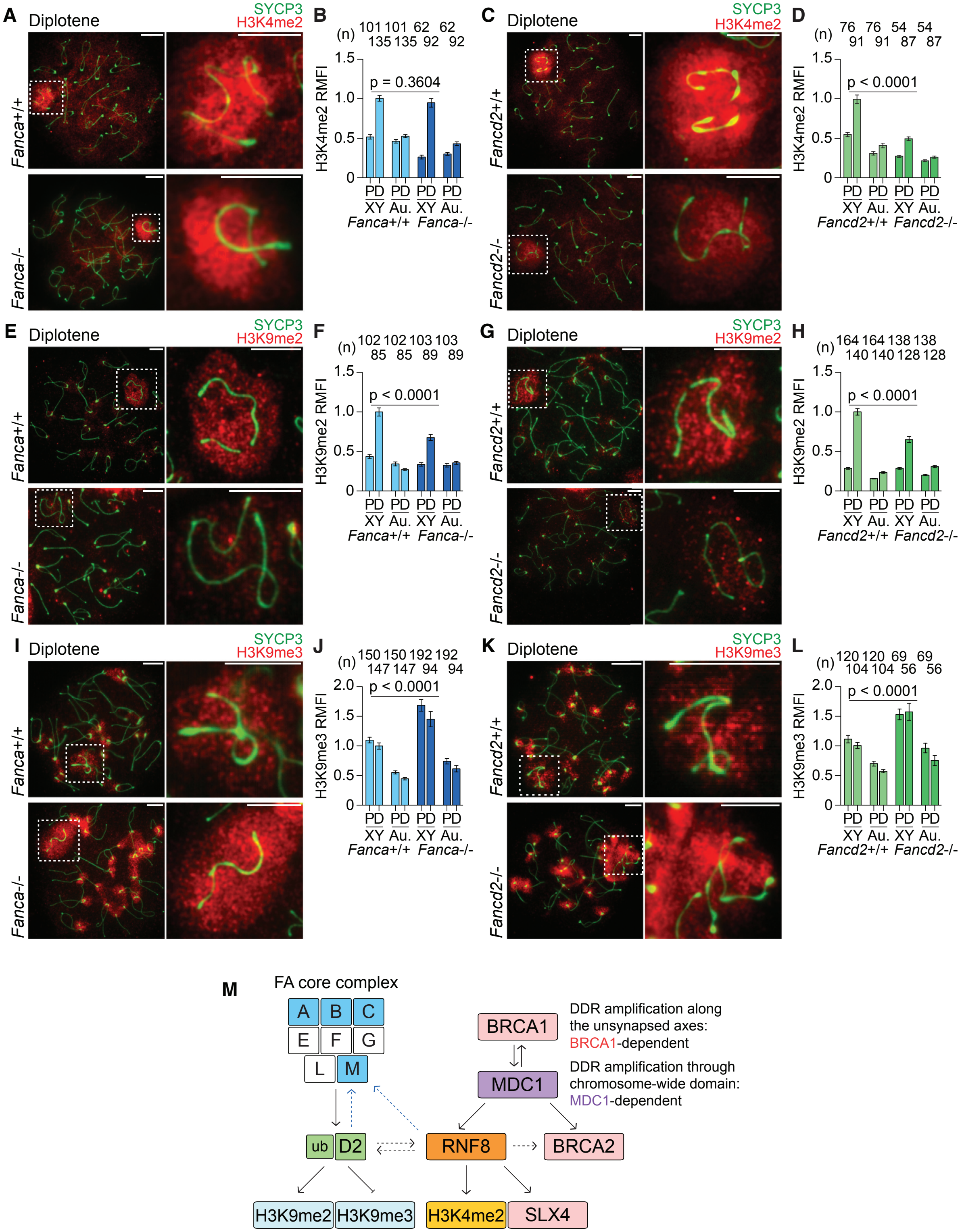

Figure 7. FANCD2 Regulates H3K4me2 Independently of FA Core Factors, whereas FA Core Factors and FANCD2 Cooperate to Regulate H3K9 Methylation

(A, C, E, G, I, and K) Immunostains using indicated antibodies in meiotic chromosome spreads from *Fanca*$^{-/-}$ mice, *Fancd2*$^{-/-}$ mice, and corresponding wild-type littermate controls. Stages are labeled above, genotypes are labeled to the left. Dashed squares border sex chromosomes and are magnified to the right. Scale bars, 5 μm.

(legend continued on next page)

the FA core complex is required for the regulation of H3K9 methylation, while the regulation of H3K4me2 is independent of the FA core complex.

These findings led us to examine the transcriptional status of the meiotic sex chromosomes in *Fancd2*$^{-/-}$ spermatocytes, a proxy for loss of function of the FA pathway. First, we performed RNA fluorescence in situ hybridization (FISH) using Cot-1 DNA probes. Cot-1 DNA probes consist of repetitive elements that can hybridize nascent transcripts, enabling the visualization of transcriptionally active regions (Hall et al., 2002; Namekawa et al., 2006, 2010). Our data revealed no significant changes in Cot-1 visualization (Figure S7A): Cot-1 was largely excluded from the XY chromatin in both control and *Fancd2*$^{-/-}$ spermatocytes, suggesting that the global transcription level is comparable between control and mutant spermatocytes. As a complementary approach to visualizing the transcriptional status of the sex chromosomes, we performed immunolocalization experiments for RNA Polymerase II (RNAPII) in *Fancd2*$^{-/-}$ spermatocytes and controls. Similar to Cot-1, RNAPII was largely excluded from the XY chromatin in both control and *Fancd2*$^{-/-}$ spermatocytes (Figure S7B). These data suggest that the initiation of MSCI is not perturbed in mutants deficient for FANCD2, although we were not able to define the transcriptional status of individual genes due to the limited numbers of *Fancd2*$^{-/-}$ spermatocytes.

As a whole, our findings define the FA-DDR network in meiosis, yield insights into the recruitment of FA proteins, and illuminate the male sex chromosomes as a model to dissect the broad FA-BRCA pathway.

DISCUSSION

In this study, we have defined how the FA-DDR network is coordinated during meiosis. By comparing mutants for FA core proteins and FANCD2, we revealed core-dependent and -independent functions of the FA pathway on meiotic sex chromosomes. In particular, we uncovered a role for the FA core complex in the accumulation of FANCD2 foci during meiosis. This is parallel to the role of the FA pathway in regulating FANCD2 foci in the somatic DDR (Garcia-Higuera et al., 2001; Taniguchi et al., 2002). However, the FA core complex is dispensable for the recruitment of BRCA2 and SLX4 to the sex chromosomes, so FA proteins do not appear to be recruited to sex chromosomes in meiosis through a simple linear pathway. And although a previous report demonstrated the necessity for FANCD2 in the recruitment of SLX4 to DNA interstrand crosslinks in somatic cells (Yamamoto et al., 2011), our results reveal FANCD2-independent recruitment of SLX4 to the XY chromatin in meiosis. Although there can be distinct mechanisms in somatic cells versus the meiotic sex chromosomes, one notable feature of the meiotic sex chromosomes is that upstream factors tend to present on axes and downstream factors tend to present on XY chromatin. In this regard, the differential localization of RAD51 and BRCA2—two factors reported to associate with each other in the canonical somatic DDR—suggests distinct regulation of each factor. In accord with this notion, in somatic cells, there is evidence of BRCA2-independent, non-canonical regulation of RAD51 recruitment to damaged chromatin (Yata et al., 2012).

Previously, we found that the focal accumulation of FANCB, an FA core protein, on the XY chromosomes is dependent on MDC1 (Kato et al., 2015). Interestingly, we find here that initial FANCD2 foci are present in *Mdc1*$^{-/-}$ spermatocytes, but that MDC1 is required for the amplification of FANCD2 foci. Thus, focal accumulation of FANCB may not be required for initial FANCD2 foci formation. In this context, it should be noted that there may be some independence in the recruitment of FA core complex proteins since they can form distinct subcomplexes (Medhurst et al., 2006).

Our data indicate that FANCM accumulation on the sex chromosomes is independent of FA core complex proteins. Yet strikingly, we report that FANCM accumulation and maintenance on the sex chromosomes is dependent on FANCD2 and the BRCA1-MDC1-RNF8 signaling axis. FANCM is an evolutionarily conserved helicase and mammalian ortholog of the archaeal DDR factor Hef (Meetei et al., 2005), and indeed, FANCM, but not the other FA core factors, has an evolutionarily conserved role in suppressing meiotic crossover recombination and directs non-crossover recombination (Crismani et al., 2012; Lorenz et al., 2012). Therefore, these reports underscore an essential and ancient function of FANCM in meiosis that is likely to be independent of the FA core complex. While FANCM has a role in promoting FANCD2 monoubiquitination and localization in the somatic DDR (Singh et al., 2009), we expect that FANCM functions with the BRCA1-MDC1-RNF8 axis independent of the FA core complex to regulate the broad FA-DDR network in meiosis.

By using mutants from the BRCA1-MDC1-RNF8 signaling axis, we show that BRCA1, MDC1, and RNF8 regulate the recruitment of BRCA2 and SLX4. In contrast, rather than having a role in the recruitment of FANCD2 to the XY axes, BRCA1 and MDC1 are instead involved in the amplification of FANCD2 signals on the sex chromosomes, as is RNF8. Notably, we previously observed a BRCA1-dependent signal amplification of RAD51 foci (Broering et al., 2014) that is similar to the amplification of FANCD2 foci found in this study. This suggests that FANCD2 and RAD51 foci may be amplified similarly along the XY axes. Consistent with this possibility, a recent study identified *Rad51* as the FA gene *Fancr* (Wang et al., 2015). Given the possible recruitment of RAD51 to sites of unrepaired double-strand breaks (DSBs) on the XY axes (Inagaki et al., 2010), initial signals of FANCD2 and RAD51 may represent sites of unrepaired DSBs. Indeed, our results demonstrate that the initial signals for

(B, D, F, H, J, and L) Quantification of H3K4me2 (B and D), H3K9me2 (F and H), and H3K9me3 (J and L) relative mean fluorescence intensity (RMFI) on sex chromosomes (XY) and autosome regions (Au.) in pachytene (P) and diplotene (D) spermatocytes. Numbers of spermatocytes analyzed are noted above each graph. Bars represent means and SEMs. Data are aggregated from n = 4 *Fanca* littermate pairs (B, F, and J), and n = 3 *Fancd2* littermate pairs (D, H, and L). p values, indicated in the panels, are derived from one-way ANOVA and Tukey's method posttest.
(M) Model of the FA-DDR network on the sex chromosomes. See the text for details.
See also Figures S6 and S7.

FANCD2 foci colocalize with RAD51 foci on XY axes in normal meiosis. Our analyses of *Spo11*$^{-/-}$ spermatocytes further support this assertion since FANCD2 colocalizes with RAD51 at SPO11-independent DNA repair foci.

FANCD2 and RAD51 signals may be amplified at undetermined sites regulated by BRCA1-MDC1-RNF8 signaling along the XY axes. These data suggest that BRCA1, MDC1, and RNF8 work as a single pathway in the regulation of FA proteins (Figure 7M). A clue to understand these undetermined sites may be inferred by studies of somatic cells. A recent proteomics analysis identified DDR protein recruitment to DNA interstrand crosslinks (Räschle et al., 2015). Because the proteins in the FA-DDR network are crucial for the repair of DNA interstrand crosslinks, it would be intriguing to compare the factors commonly present both at meiotic sex chromosomes and DNA interstrand crosslinks in somatic cells.

Here, by investigating meiosis, we demonstrate a critical role for RNF8 in the regulation of four different FA proteins: FANCM, FANCD2, BRCA2, and SLX4 (Figure 7M). One possible mechanism by which RNF8 regulates FA proteins is RNF8-mediated ubiquitination established on the XY chromatin for subsequent epigenetic programming (Sin et al., 2012). In support of this possibility, SLX4 binds ubiquitinated substrates (Yamamoto et al., 2011). This may help to explain our finding that SLX4 recruitment to XY chromatin is RNF8 dependent. In turn, we demonstrate here that FANCD2, but not FA core proteins, modulates H3K4me2, an RNF8-dependent modification, suggesting a possible role for FANCD2 in RNF8-dependent epigenetic programming. The functional link between FANCD2 and RNF8 is further supported by the fact that both factors regulate FANCM. Based on these results, we propose a model in which FANCD2 and RNF8 function together, serving as a central link between the FA pathway and the BRCA1-MDC1-RNF8 signaling axis to integrate the FA-DDR network (Figure 7M).

Unlike H3K4me2, H3K9 methylation is regulated by FA core factors. Thus, there are both core-dependent and -independent roles for FANCD2 in meiotic prophase. A previous report showed that germ cells and testicular size were severely compromised in *Fancd2*$^{-/-}$ mice (Houghtaling et al., 2003) as compared with other mutants of FA core subunits (including *Fanca*$^{-/-}$ and *Fancc*$^{-/-}$) that display subfertility (Whitney et al., 1996; Wong et al., 2003). This severe germline phenotype of *Fancd2*$^{-/-}$ mice could indicate additional roles for FANCD2 beyond its canonical function in the FA pathway downstream of the FA core complex. The different roles for FANCD2 and the FA core complex in epigenetic regulation could be related to the fact that the localization of FANCD2 is regulated by both the FA core complex and by RNF8. FANCD2 foci require the FA core complex and the FA pathway may thereby be involved in the regulation of H3K9 methylation.

In contrast, the role of FANCD2 in the regulation of H3K4me2 levels does not appear to be dependent on its monoubiquitination since the FA core complex is not involved in regulating H3K4me2. But RNF8 also has some control over FANCD2 foci, which could be related to FA core-independent regulation of H3K4me2 levels. To clarify the molecular mechanisms that underlie these differences, it will be important to dissect the molecular link between FA proteins and epigenetic programming in future studies. We recently demonstrated that the substrate of RNF8-mediated ubiquitination on XY chromatin is an unknown target that is not histone H2A (Hasegawa et al., 2015). While this ubiquitin substrate of RNF8 had a different size and is therefore not likely to be FANCD2, it will be important to identify this substrate and interesting to test whether it is regulated by FANCD2. Intriguingly, the function of FANCD2 in epigenetic programming of the sex chromosomes concurs with the recent finding that FANCD2 has histone chaperone activity in DNA crosslink repair (Sato et al., 2012). Because the initiation of MSCI is followed by the replacement of histone H3.3 (van der Heijden et al., 2007), it is conceivable that FANCD2 is involved in this step to regulate epigenetic programming.

Taken together, these results demonstrate that the FA proteins, together with MDC1 and RNF8, comprise the FA-DDR network, which governs the sex chromosomes during meiosis (Figure 7M). This raises the possibility that common pathways underlie both the regulation of sex chromosomes during meiosis and the somatic DDR. Therefore, MSCI has emerged as a model system to dissect the roles of different FA proteins in the DDR and in epigenetic programming, and for understanding how FA proteins are regulated and how they are interrelated. In future studies, the coordinated spatial and temporal localization of FA proteins on the meiotic sex chromosomes will enable us to use FA genetic models to dissect the details of the FA-BRCA pathway and subsequent epigenetic programming in high resolution. Furthermore, the functional consequence of histone modification changes observed in FA mutant mice has emerged as another important area of investigation. Because RNF8 is required for establishing active modifications and gene activation of male reproduction genes from inactive sex chromosomes in spermatids (Sin et al., 2012), it is possible that the changes in histone modification may alter individual gene expression from silent sex chromosomes in FA mutant mice. Understanding the role of FA proteins in epigenetic programming may also be important for understanding fertility defects associated with FA, and for understanding the role and regulation of FA proteins in DNA repair.

EXPERIMENTAL PROCEDURES

Mice

Fanca$^{-/-}$, *Fancb*$^{-/Y}$, *Fancc*$^{-/-}$, *Fancd2*$^{-/-}$, *Mdc1*$^{-/-}$, *Rnf8*$^{-/-}$, and *Spo11*$^{-/-}$ mouse models were previously described (Chen et al., 1996; Cheng et al., 2000; Houghtaling et al., 2003; Kato et al., 2015; Lou et al., 2006; Minter-Dykhouse et al., 2008; Romanienko and Camerini-Otero, 2000). Mice with a conditional deletion of *Brca1* exon 11 using *Ddx4*-cre were previously described (Broering et al., 2014). *Rnf8*$^{-/-}$, *Mdc1*$^{-/-}$, *Fancb*$^{-/Y}$, and *Spo11*$^{-/-}$ mouse models were on C57BL/6 backgrounds; *Fanca*$^{-/-}$, *Fancc*$^{-/-}$, *Fancd2*$^{-/-}$, and *Brca1*cKO mouse models were on mixed backgrounds. This work was approved by Institutional Animal Care and Use Committee protocol no. IACUC2015-0032.

Slide Preparation and Cytological Analyses

Meiotic chromosomes were analyzed with surface spreads prepared via hypotonic treatment, modified from an established protocol (Peters et al., 1997). Immunofluorescence was performed as described (Kato et al., 2015). Specialized slides that preserve the relative three-dimensional nuclear architecture of testicular germ cells were prepared as described (Namekawa et al., 2006, Namekawa, 2014; Namekawa and Lee, 2011). Cot-1 RNA FISH was performed as described (Namekawa and Lee, 2011).

Statistical Analyses

Means were compared using unpaired two-tailed Student's t tests for experiments involving two groups. Two-tailed one-way ANOVA was used when comparisons were made across more than two groups, and Tukey's method was performed as a posttest for pairwise comparisons. Pearson's chi-square test was used to identify categorical differences in the accumulation of BRCA2 (Figure 4) and FANCM (Figures 6 and S4) between two groups. Unless specified, p values are indicated with text or asterisks as follows: n.s., not significant, $p > 0.05$; *$p \leq 0.05$; **$p \leq 0.01$; ***$p \leq 0.001$.

SUPPLEMENTAL INFORMATION

Supplemental Information includes Supplemental Experimental Procedures and seven figures and can be found with this article online at http://dx.doi.org/10.1016/j.celrep.2016.09.073.

AUTHOR CONTRIBUTIONS

K.G.A., P.R.A., and S.H.N. designed the experiments. K.G.A. performed and interpreted most of the experiments. Y.K., H.-S.S., S.M., I.J.K., F.Z., and S.H.N. contributed experiments. Q.P. and P.R.A. provided materials. K.G.A., P.R.A., and S.H.N. wrote the manuscript with all other authors.

ACKNOWLEDGMENTS

We thank past and present members of S.H.N.'s lab, P.R.A.'s lab, and the Division of Reproductive Sciences for discussion and helpful comments regarding the manuscript; Madeleine Carreau for providing *Fanca*$^{+/-}$ mice; Manuel Buchwald for providing *Fancc*$^{+/-}$ mice; Markus Grompe for providing *Fancd2*$^{+/-}$ mice; Junjie Chen for providing *Mdc1*$^{+/-}$ and *Rnf8*$^{+/-}$ mice; R. Daniel Camerini-Otero for *Spo11*$^{+/-}$ mice; the Fanconi Anemia Research Fund for the FANCM antibody; Alan D'Andrea for FANCD2 antibodies (G33 and E35); and Paula E. Cohen for the SLX4 antibody. This work was supported by the Research Grant (FY13-510) from the March of Dimes Foundation to S.H.N. and NIH grants (HL085587 to P.R.A. and GM098605 to S.H.N.).

Received: December 18, 2015
Revised: August 17, 2016
Accepted: September 21, 2016
Published: October 18, 2016

REFERENCES

An, J.Y., Kim, E., Zakrzewska, A., Yoo, Y.D., Jang, J.M., Han, D.H., Lee, M.J., Seo, J.W., Lee, Y.J., Kim, T.Y., et al. (2012). UBR2 of the N-end rule pathway is required for chromosome stability via histone ubiquitylation in spermatocytes and somatic cells. PLoS ONE *7*, e37414.

Bakker, S.T., van de Vrugt, H.J., Rooimans, M.A., Oostra, A.B., Steltenpool, J., Delzenne-Goette, E., van der Wal, A., van der Valk, M., Joenje, H., te Riele, H., and de Winter, J.P. (2009). Fancm-deficient mice reveal unique features of Fanconi anemia complementation group M. Hum. Mol. Genet. *18*, 3484–3495.

Barchi, M., Mahadevaiah, S., Di Giacomo, M., Baudat, F., de Rooij, D.G., Burgoyne, P.S., Jasin, M., and Keeney, S. (2005). Surveillance of different recombination defects in mouse spermatocytes yields distinct responses despite elimination at an identical developmental stage. Mol. Cell. Biol. *25*, 7203–7215.

Baudat, F., Manova, K., Yuen, J.P., Jasin, M., and Keeney, S. (2000). Chromosome synapsis defects and sexually dimorphic meiotic progression in mice lacking Spo11. Mol. Cell *6*, 989–998.

Bellani, M.A., Romanienko, P.J., Cairatti, D.A., and Camerini-Otero, R.D. (2005). SPO11 is required for sex-body formation, and Spo11 heterozygosity rescues the prophase arrest of Atm-/- spermatocytes. J. Cell Sci. *118*, 3233–3245.

Bick, G., Zhang, F., Meetei, A.R., and Andreassen, P.R. (2016). Coordination of the recruitment of the FANCD2 and PALB2 Fanconi anemia proteins by an ubiquitin signaling network. Chromosoma, Published online June 8, 2016. http://dx.doi.org/10.1007/s00412-016-0602-9.

Bluteau, D., Masliah-Planchon, J., Clairmont, C., Rousseau, A., Ceccaldi, R., Dubois d'Enghien, C., Bluteau, O., Cuccuini, W., Gachet, S., Peffault de Latour, R., et al. (2016). Biallelic inactivation of REV7 is associated with Fanconi anemia. J. Clin. Invest. *126*, 3580–3584.

Broering, T.J., Alavattam, K.G., Sadreyev, R.I., Ichijima, Y., Kato, Y., Hasegawa, K., Camerini-Otero, R.D., Lee, J.T., Andreassen, P.R., and Namekawa, S.H. (2014). BRCA1 establishes DNA damage signaling and pericentric heterochromatin of the X chromosome in male meiosis. J. Cell Biol. *205*, 663–675.

Carofiglio, F., Inagaki, A., de Vries, S., Wassenaar, E., Schoenmakers, S., Vermeulen, C., van Cappellen, W.A., Sleddens-Linkels, E., Grootegoed, J.A., Te Riele, H.P., et al. (2013). SPO11-independent DNA repair foci and their role in meiotic silencing. PLoS Genet. *9*, e1003538.

Chen, M., Tomkins, D.J., Auerbach, W., McKerlie, C., Youssoufian, H., Liu, L., Gan, O., Carreau, M., Auerbach, A., Groves, T., et al. (1996). Inactivation of Fac in mice produces inducible chromosomal instability and reduced fertility reminiscent of Fanconi anaemia. Nat. Genet. *12*, 448–451.

Chen, J., Silver, D.P., Walpita, D., Cantor, S.B., Gazdar, A.F., Tomlinson, G., Couch, F.J., Weber, B.L., Ashley, T., Livingston, D.M., and Scully, R. (1998). Stable interaction between the products of the BRCA1 and BRCA2 tumor suppressor genes in mitotic and meiotic cells. Mol. Cell *2*, 317–328.

Cheng, N.C., van de Vrugt, H.J., van der Valk, M.A., Oostra, A.B., Krimpenfort, P., de Vries, Y., Joenje, H., Berns, A., and Arwert, F. (2000). Mice with a targeted disruption of the Fanconi anemia homolog Fanca. Hum. Mol. Genet. *9*, 1805–1811.

Ciccia, A., and Elledge, S.J. (2010). The DNA damage response: making it safe to play with knives. Mol. Cell *40*, 179–204.

Crismani, W., Girard, C., Froger, N., Pradillo, M., Santos, J.L., Chelysheva, L., Copenhaver, G.P., Horlow, C., and Mercier, R. (2012). FANCM limits meiotic crossovers. Science *336*, 1588–1590.

Fernandez-Capetillo, O., Mahadevaiah, S.K., Celeste, A., Romanienko, P.J., Camerini-Otero, R.D., Bonner, W.M., Manova, K., Burgoyne, P., and Nussenzweig, A. (2003). H2AX is required for chromatin remodeling and inactivation of sex chromosomes in male mouse meiosis. Dev. Cell *4*, 497–508.

Folias, A., Matkovic, M., Bruun, D., Reid, S., Hejna, J., Grompe, M., D'Andrea, A., and Moses, R. (2002). BRCA1 interacts directly with the Fanconi anemia protein FANCA. Hum. Mol. Genet. *11*, 2591–2597.

Gallardo, T., Shirley, L., John, G.B., and Castrillon, D.H. (2007). Generation of a germ cell-specific mouse transgenic Cre line, Vasa-Cre. Genesis *45*, 413–417.

Garcia-Higuera, I., Taniguchi, T., Ganesan, S., Meyn, M.S., Timmers, C., Hejna, J., Grompe, M., and D'Andrea, A.D. (2001). Interaction of the Fanconi anemia proteins and BRCA1 in a common pathway. Mol. Cell *7*, 249–262.

Goldberg, M., Stucki, M., Falck, J., D'Amours, D., Rahman, D., Pappin, D., Bartek, J., and Jackson, S.P. (2003). MDC1 is required for the intra-S-phase DNA damage checkpoint. Nature *421*, 952–956.

Greaves, I.K., Rangasamy, D., Devoy, M., Marshall Graves, J.A., and Tremethick, D.J. (2006). The X and Y chromosomes assemble into H2A.Z-containing [corrected] facultative heterochromatin [corrected] following meiosis. Mol. Cell. Biol. *26*, 5394–5405.

Hall, L.L., Byron, M., Sakai, K., Carrel, L., Willard, H.F., and Lawrence, J.B. (2002). An ectopic human XIST gene can induce chromosome inactivation in postdifferentiation human HT-1080 cells. Proc. Natl. Acad. Sci. USA *99*, 8677–8682.

Hasegawa, K., Sin, H.S., Maezawa, S., Broering, T.J., Kartashov, A.V., Alavattam, K.G., Ichijima, Y., Zhang, F., Bacon, W.C., Greis, K.D., et al. (2015). SCML2 establishes the male germline epigenome through regulation of histone H2A ubiquitination. Dev. Cell *32*, 574–588.

Holloway, J.K., Mohan, S., Balmus, G., Sun, X., Modzelewski, A., Borst, P.L., Freire, R., Weiss, R.S., and Cohen, P.E. (2011). Mammalian BTBD12 (SLX4) protects against genomic instability during mammalian spermatogenesis. PLoS Genet. *7*, e1002094.

Houghtaling, S., Timmers, C., Noll, M., Finegold, M.J., Jones, S.N., Meyn, M.S., and Grompe, M. (2003). Epithelial cancer in Fanconi anemia complementation group D2 (Fancd2) knockout mice. Genes Dev. *17*, 2021–2035.

Howlett, N.G., Taniguchi, T., Olson, S., Cox, B., Waisfisz, Q., De Die-Smulders, C., Persky, N., Grompe, M., Joenje, H., Pals, G., et al. (2002). Biallelic inactivation of BRCA2 in Fanconi anemia. Science *297*, 606–609.

Huen, M.S., Grant, R., Manke, I., Minn, K., Yu, X., Yaffe, M.B., and Chen, J. (2007). RNF8 transduces the DNA-damage signal via histone ubiquitylation and checkpoint protein assembly. Cell *131*, 901–914.

Ichijima, Y., Ichijima, M., Lou, Z., Nussenzweig, A., Camerini-Otero, R.D., Chen, J., Andreassen, P.R., and Namekawa, S.H. (2011). MDC1 directs chromosome-wide silencing of the sex chromosomes in male germ cells. Genes Dev. *25*, 959–971.

Ichijima, Y., Sin, H.S., and Namekawa, S.H. (2012). Sex chromosome inactivation in germ cells: emerging roles of DNA damage response pathways. Cell. Mol. Life Sci. *69*, 2559–2572.

Inagaki, A., Schoenmakers, S., and Baarends, W.M. (2010). DNA double strand break repair, chromosome synapsis and transcriptional silencing in meiosis. Epigenetics *5*, 255–266.

Kato, Y., Alavattam, K.G., Sin, H.S., Meetei, A.R., Pang, Q., Andreassen, P.R., and Namekawa, S.H. (2015). FANCB is essential in the male germline and regulates H3K9 methylation on the sex chromosomes during meiosis. Hum. Mol. Genet. *24*, 5234–5249.

Kee, Y., and D'Andrea, A.D. (2010). Expanded roles of the Fanconi anemia pathway in preserving genomic stability. Genes Dev. *24*, 1680–1694.

Khalil, A.M., Boyar, F.Z., and Driscoll, D.J. (2004). Dynamic histone modifications mark sex chromosome inactivation and reactivation during mammalian spermatogenesis. Proc. Natl. Acad. Sci. USA *101*, 16583–16587.

Kim, Y., Lach, F.P., Desetty, R., Hanenberg, H., Auerbach, A.D., and Smogorzewska, A. (2011). Mutations of the SLX4 gene in Fanconi anemia. Nat. Genet. *43*, 142–146.

Kolas, N.K., Chapman, J.R., Nakada, S., Ylanko, J., Chahwan, R., Sweeney, F.D., Panier, S., Mendez, M., Wildenhain, J., Thomson, T.M., et al. (2007). Orchestration of the DNA-damage response by the RNF8 ubiquitin ligase. Science *318*, 1637–1640.

Kottemann, M.C., and Smogorzewska, A. (2013). Fanconi anaemia and the repair of Watson and Crick DNA crosslinks. Nature *493*, 356–363.

Lachaud, C., Castor, D., Hain, K., Muñoz, I., Wilson, J., MacArtney, T.J., Schindler, D., and Rouse, J. (2014). Distinct functional roles for the two SLX4 ubiquitin-binding UBZ domains mutated in Fanconi anemia. J. Cell Sci. *127*, 2811–2817.

Lorenz, A., Osman, F., Sun, W., Nandi, S., Steinacher, R., and Whitby, M.C. (2012). The fission yeast FANCM ortholog directs non-crossover recombination during meiosis. Science *336*, 1585–1588.

Lou, Z., Minter-Dykhouse, K., Wu, X., and Chen, J. (2003). MDC1 is coupled to activated CHK2 in mammalian DNA damage response pathways. Nature *421*, 957–961.

Lou, Z., Minter-Dykhouse, K., Franco, S., Gostissa, M., Rivera, M.A., Celeste, A., Manis, J.P., van Deursen, J., Nussenzweig, A., Paull, T.T., et al. (2006). MDC1 maintains genomic stability by participating in the amplification of ATM-dependent DNA damage signals. Mol. Cell *21*, 187–200.

Lu, L.Y., Xiong, Y., Kuang, H., Korakavi, G., and Yu, X. (2013). Regulation of the DNA damage response on male meiotic sex chromosomes. Nat. Commun. *4*, 2105.

Mailand, N., Bekker-Jensen, S., Faustrup, H., Melander, F., Bartek, J., Lukas, C., and Lukas, J. (2007). RNF8 ubiquitylates histones at DNA double-strand breaks and promotes assembly of repair proteins. Cell *131*, 887–900.

Medhurst, A.L., Laghmani, H., Steltenpool, J., Ferrer, M., Fontaine, C., de Groot, J., Rooimans, M.A., Scheper, R.J., Meetei, A.R., Wang, W., et al. (2006). Evidence for subcomplexes in the Fanconi anemia pathway. Blood *108*, 2072–2080.

Meetei, A.R., de Winter, J.P., Medhurst, A.L., Wallisch, M., Waisfisz, Q., van de Vrugt, H.J., Oostra, A.B., Yan, Z., Ling, C., Bishop, C.E., et al. (2003). A novel ubiquitin ligase is deficient in Fanconi anemia. Nat. Genet. *35*, 165–170.

Meetei, A.R., Medhurst, A.L., Ling, C., Xue, Y., Singh, T.R., Bier, P., Steltenpool, J., Stone, S., Dokal, I., Mathew, C.G., et al. (2005). A human ortholog of archaeal DNA repair protein Hef is defective in Fanconi anemia complementation group M. Nat. Genet. *37*, 958–963.

Minter-Dykhouse, K., Ward, I., Huen, M.S., Chen, J., and Lou, Z. (2008). Distinct versus overlapping functions of MDC1 and 53BP1 in DNA damage response and tumorigenesis. J. Cell Biol. *181*, 727–735.

Moynahan, M.E., Pierce, A.J., and Jasin, M. (2001). BRCA2 is required for homology-directed repair of chromosomal breaks. Mol. Cell *7*, 263–272.

Namekawa, S.H. (2014). Slide preparation method to preserve three-dimensional chromatin architecture of testicular germ cells. J. Vis. Exp. *10*, e50819.

Namekawa, S.H., and Lee, J.T. (2011). Detection of nascent RNA, single-copy DNA and protein localization by immunoFISH in mouse germ cells and preimplantation embryos. Nat. Protoc. *6*, 270–284.

Namekawa, S.H., Park, P.J., Zhang, L.F., Shima, J.E., McCarrey, J.R., Griswold, M.D., and Lee, J.T. (2006). Postmeiotic sex chromatin in the male germline of mice. Curr. Biol. *16*, 660–667.

Namekawa, S.H., Payer, B., Huynh, K.D., Jaenisch, R., and Lee, J.T. (2010). Two-step imprinted X inactivation: repeat versus genic silencing in the mouse. Mol. Cell. Biol. *30*, 3187–3205.

Park, J.Y., Singh, T.R., Nassar, N., Zhang, F., Freund, M., Hanenberg, H., Meetei, A.R., and Andreassen, P.R. (2014a). Breast cancer-associated missense mutants of the PALB2 WD40 domain, which directly binds RAD51C, RAD51 and BRCA2, disrupt DNA repair. Oncogene *33*, 4803–4812.

Park, J.Y., Zhang, F., and Andreassen, P.R. (2014b). PALB2: the hub of a network of tumor suppressors involved in DNA damage responses. Biochim. Biophys. Acta *1846*, 263–275.

Park, J.Y., Virts, E.L., Jankowska, A., Wiek, C., Othman, M., Chakraborty, S.C., Vance, G.H., Alkuraya, F.S., Hanenberg, H., and Andreassen, P.R. (2016). Complementation of hypersensitivity to DNA interstrand crosslinking agents demonstrates that XRCC2 is a Fanconi anaemia gene. J. Med. Genet. *53*, 672–680.

Peters, A.H., Plug, A.W., van Vugt, M.J., and de Boer, P. (1997). A drying-down technique for the spreading of mammalian meiocytes from the male and female germline. Chromosome Res. *5*, 66–68.

Polo, S.E., and Jackson, S.P. (2011). Dynamics of DNA damage response proteins at DNA breaks: a focus on protein modifications. Genes Dev. *25*, 409–433.

Räschle, M., Smeenk, G., Hansen, R.K., Temu, T., Oka, Y., Hein, M.Y., Nagaraj, N., Long, D.T., Walter, J.C., Hofmann, K., et al. (2015). DNA repair. Proteomics reveals dynamic assembly of repair complexes during bypass of DNA cross-links. Science *348*, 1253671.

Romanienko, P.J., and Camerini-Otero, R.D. (2000). The mouse Spo11 gene is required for meiotic chromosome synapsis. Mol. Cell *6*, 975–987.

Royo, H., Prosser, H., Ruzankina, Y., Mahadevaiah, S.K., Cloutier, J.M., Baumann, M., Fukuda, T., Höög, C., Tóth, A., de Rooij, D.G., et al. (2013). ATR acts stage specifically to regulate multiple aspects of mammalian meiotic silencing. Genes Dev. *27*, 1484–1494.

Sato, K., Ishiai, M., Toda, K., Furukoshi, S., Osakabe, A., Tachiwana, H., Takizawa, Y., Kagawa, W., Kitao, H., Dohmae, N., et al. (2012). Histone chaperone activity of Fanconi anemia proteins, FANCD2 and FANCI, is required for DNA crosslink repair. EMBO J. *31*, 3524–3536.

Sawyer, S.L., Tian, L., Kähkönen, M., Schwartzentruber, J., Kircher, M., Majewski, J., Dyment, D.A., Innes, A.M., Boycott, K.M., Moreau, L.A., et al.; University of Washington Centre for Mendelian Genomics; FORGE Canada Consortium (2015). Biallelic mutations in BRCA1 cause a new Fanconi anemia subtype. Cancer Discov. *5*, 135–142.

Sims, A.E., Spiteri, E., Sims, R.J., 3rd, Arita, A.G., Lach, F.P., Landers, T., Wurm, M., Freund, M., Neveling, K., Hanenberg, H., et al. (2007). FANCI is a second monoubiquitinated member of the Fanconi anemia pathway. Nat. Struct. Mol. Biol. *14*, 564–567.

Sin, H.S., Barski, A., Zhang, F., Kartashov, A.V., Nussenzweig, A., Chen, J., Andreassen, P.R., and Namekawa, S.H. (2012). RNF8 regulates active epigenetic modifications and escape gene activation from inactive sex chromosomes in post-meiotic spermatids. Genes Dev. *26*, 2737–2748.

Singh, T.R., Bakker, S.T., Agarwal, S., Jansen, M., Grassman, E., Godthelp, B.C., Ali, A.M., Du, C.H., Rooimans, M.A., Fan, Q., et al. (2009). Impaired FANCD2 monoubiquitination and hypersensitivity to camptothecin uniquely characterize Fanconi anemia complementation group M. Blood *114*, 174–180.

Smogorzewska, A., Matsuoka, S., Vinciguerra, P., McDonald, E.R., 3rd, Hurov, K.E., Luo, J., Ballif, B.A., Gygi, S.P., Hofmann, K., D'Andrea, A.D., and Elledge, S.J. (2007). Identification of the FANCI protein, a monoubiquitinated FANCD2 paralog required for DNA repair. Cell *129*, 289–301.

Stewart, G.S., Wang, B., Bignell, C.R., Taylor, A.M., and Elledge, S.J. (2003). MDC1 is a mediator of the mammalian DNA damage checkpoint. Nature *421*, 961–966.

Stoepker, C., Hain, K., Schuster, B., Hilhorst-Hofstee, Y., Rooimans, M.A., Steltenpool, J., Oostra, A.B., Eirich, K., Korthof, E.T., Nieuwint, A.W., et al. (2011). SLX4, a coordinator of structure-specific endonucleases, is mutated in a new Fanconi anemia subtype. Nat. Genet. *43*, 138–141.

Taniguchi, T., Garcia-Higuera, I., Andreassen, P.R., Gregory, R.C., Grompe, M., and D'Andrea, A.D. (2002). S-phase-specific interaction of the Fanconi anemia protein, FANCD2, with BRCA1 and RAD51. Blood *100*, 2414–2420.

Timmers, C., Taniguchi, T., Hejna, J., Reifsteck, C., Lucas, L., Bruun, D., Thayer, M., Cox, B., Olson, S., D'Andrea, A.D., et al. (2001). Positional cloning of a novel Fanconi anemia gene, FANCD2. Mol. Cell *7*, 241–248.

Turner, J.M. (2007). Meiotic sex chromosome inactivation. Development *134*, 1823–1831.

Turner, J.M., Aprelikova, O., Xu, X., Wang, R., Kim, S., Chandramouli, G.V., Barrett, J.C., Burgoyne, P.S., and Deng, C.X. (2004). BRCA1, histone H2AX phosphorylation, and male meiotic sex chromosome inactivation. Curr. Biol. *14*, 2135–2142.

Turner, J.M., Mahadevaiah, S.K., Ellis, P.J., Mitchell, M.J., and Burgoyne, P.S. (2006). Pachytene asynapsis drives meiotic sex chromosome inactivation and leads to substantial postmeiotic repression in spermatids. Dev. Cell *10*, 521–529.

van der Heijden, G.W., Derijck, A.A., Pósfai, E., Giele, M., Pelczar, P., Ramos, L., Wansink, D.G., van der Vlag, J., Peters, A.H., and de Boer, P. (2007). Chromosome-wide nucleosome replacement and H3.3 incorporation during mammalian meiotic sex chromosome inactivation. Nat. Genet. *39*, 251–258.

Vandenberg, C.J., Gergely, F., Ong, C.Y., Pace, P., Mallery, D.L., Hiom, K., and Patel, K.J. (2003). BRCA1-independent ubiquitination of FANCD2. Mol. Cell *12*, 247–254.

Wang, X., Andreassen, P.R., and D'Andrea, A.D. (2004). Functional interaction of monoubiquitinated FANCD2 and BRCA2/FANCD1 in chromatin. Mol. Cell. Biol. *24*, 5850–5862.

Wang, A.T., Kim, T., Wagner, J.E., Conti, B.A., Lach, F.P., Huang, A.L., Molina, H., Sanborn, E.M., Zierhut, H., Cornes, B.K., et al. (2015). A Dominant Mutation in Human RAD51 Reveals Its Function in DNA Interstrand Crosslink Repair Independent of Homologous Recombination. Mol. Cell *59*, 478–490.

Whitney, M.A., Royle, G., Low, M.J., Kelly, M.A., Axthelm, M.K., Reifsteck, C., Olson, S., Braun, R.E., Heinrich, M.C., Rathbun, R.K., et al. (1996). Germ cell defects and hematopoietic hypersensitivity to gamma-interferon in mice with a targeted disruption of the Fanconi anemia C gene. Blood *88*, 49–58.

Wong, J.C., Alon, N., Mckerlie, C., Huang, J.R., Meyn, M.S., and Buchwald, M. (2003). Targeted disruption of exons 1 to 6 of the Fanconi Anemia group A gene leads to growth retardation, strain-specific microphthalmia, meiotic defects and primordial germ cell hypoplasia. Hum. Mol. Genet. *12*, 2063–2076.

Xu, X., Aprelikova, O., Moens, P., Deng, C.X., and Furth, P.A. (2003). Impaired meiotic DNA-damage repair and lack of crossing-over during spermatogenesis in BRCA1 full-length isoform deficient mice. Development *130*, 2001–2012.

Yamamoto, K.N., Kobayashi, S., Tsuda, M., Kurumizaka, H., Takata, M., Kono, K., Jiricny, J., Takeda, S., and Hirota, K. (2011). Involvement of SLX4 in interstrand cross-link repair is regulated by the Fanconi anemia pathway. Proc. Natl. Acad. Sci. USA *108*, 6492–6496.

Yan, Z., Guo, R., Paramasivam, M., Shen, W., Ling, C., Fox, D., 3rd, Wang, Y., Oostra, A.B., Kuehl, J., Lee, D.Y., et al. (2012). A ubiquitin-binding protein, FAAP20, links RNF8-mediated ubiquitination to the Fanconi anemia DNA repair network. Mol. Cell *47*, 61–75.

Yata, K., Lloyd, J., Maslen, S., Bleuyard, J.Y., Skehel, M., Smerdon, S.J., and Esashi, F. (2012). Plk1 and CK2 act in concert to regulate Rad51 during DNA double strand break repair. Mol. Cell *45*, 371–383.

Zhang, F., Fan, Q., Ren, K., and Andreassen, P.R. (2009a). PALB2 functionally connects the breast cancer susceptibility proteins BRCA1 and BRCA2. Mol. Cancer Res. *7*, 1110–1118.

Zhang, F., Ma, J., Wu, J., Ye, L., Cai, H., Xia, B., and Yu, X. (2009b). PALB2 links BRCA1 and BRCA2 in the DNA-damage response. Curr. Biol. *19*, 524–529.

Zhang, F., Fan, Q., Ren, K., Auerbach, A.D., and Andreassen, P.R. (2010). FANCJ/BRIP1 recruitment and regulation of FANCD2 in DNA damage responses. Chromosoma *119*, 637–649.

Zhang, F., Bick, G., Park, J.Y., and Andreassen, P.R. (2012). MDC1 and RNF8 function in a pathway that directs BRCA1-dependent localization of PALB2 required for homologous recombination. J. Cell Sci. *125*, 6049–6057.

Cell Reports, Volume *17*

Supplemental Information

Elucidation of the Fanconi Anemia Protein Network in Meoisis and Its Function in the Regulation of Histone Modifications

Kris G. Alavattam, Yasuko Kato, Ho-Su Sin, So Maezawa, Ian J. Kowalski, Fan Zhang, Qishen Pang, Paul R. Andreassen, and Satoshi H. Namekawa

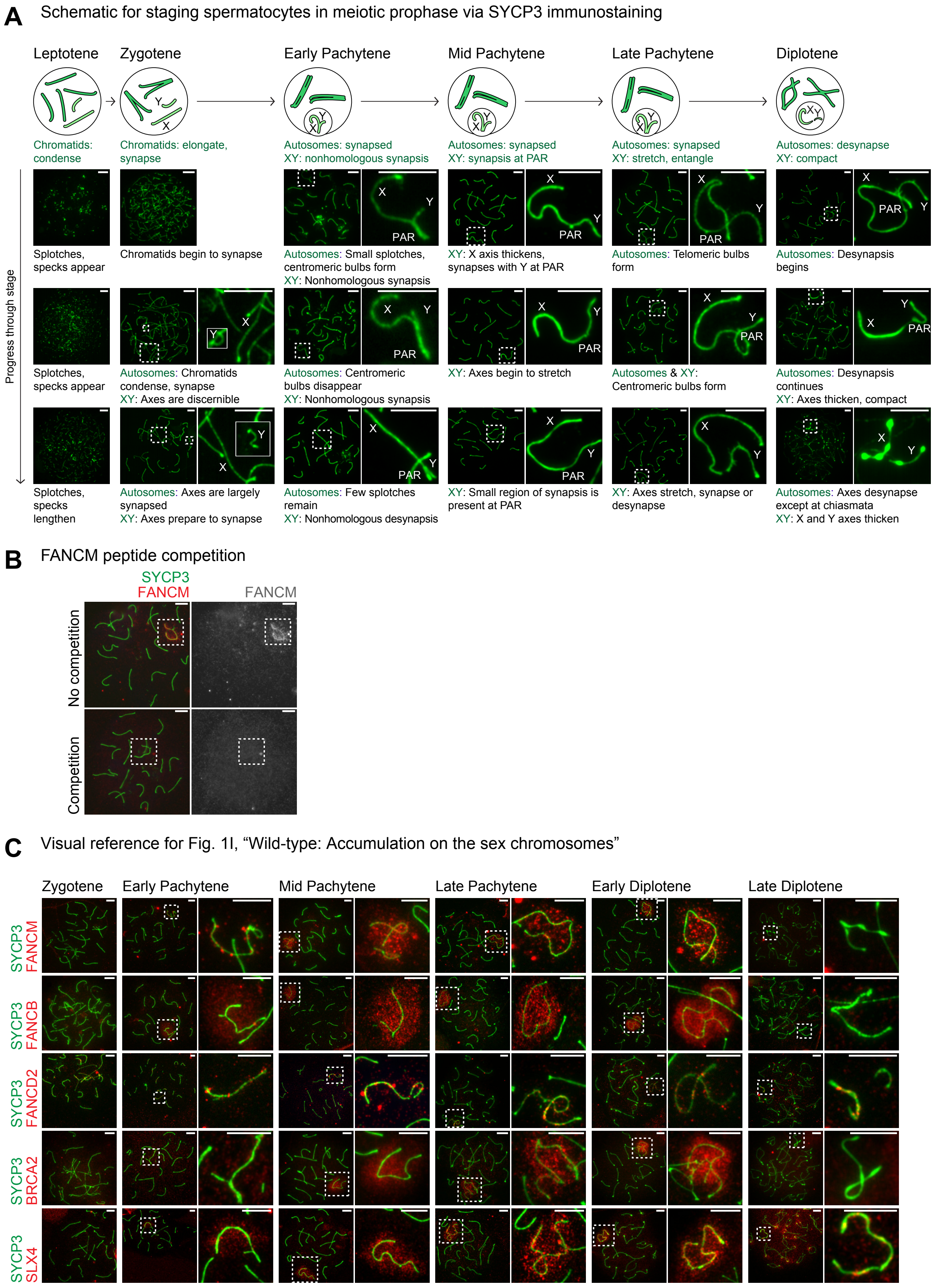

(legend on next page)

Figure S1. Cytology and criteria for staging spermatocytes in meiotic prophase via SYCP3 staining, related to Figure 1.

(**A**) Meiotic prophase is divided into four stages based on the presentation and pairing—or synapsis—of paternal and maternal chromatids, known as non-sister chromatids. Chromatids are the threadlike strands that chromosomes condense into during cell division. Essential to this condensation and synapsis is SYCP3 (shown in green), a structural component of the synaptonemal complex protein polymer. Through immunofluorescent microscopy of chromosome spreads stained with anti-SYCP3 antibody, it is possible to stage spermatocytes in meiotic prophase with a high degree of accuracy. In the first stage of meiotic prophase, the leptotene stage, maternal and paternal chromatids begin to condense and elongate. In the following stage, the zygotene stage, the chromatids continue to elongate and begin to synapse. The subsequent pachytene stage lasts the longest and, as such, is subdivided into three stages to account for its many appearances: the early, mid, and late pachytene stages. In the early pachytene stage, all chromosomes that are not sex chromosomes—that is, autosomes—have synapsed, and the male sex chromosomes, X and Y, undergo partial synapsis at a small region known as the pseudo-autosomal region (PAR). During the early pachytene stage, XY synapsis increases until most of the Y chromosome axis is nonhomologously synapsed to X; then, as spermatocytes progress through the mid and late pachytene stages, X and Y desynapse. In the next stage of meiotic prophase, the diplotene stage, X and Y compact while the autosomes progressively desynapse except at specialized regions of contact known as chiasmata, where the exchange—or recombination—of genetic material occurs between non-sister chromatids. In the panel, the progression through stages are shown top to bottom and are indicated by an arrow: top images in each column represent early examples of each stage, middle images represent intermediate examples, and bottom images represent late examples. The sex chromosomes are indicated by dashed squares, and these squares are magnified in panels to the right. In the sample cytology for the zygotene stage, magnified images of Y are inlaid with magnified images of X. X: X chromosome; Y: Y chromosome; PAR: pseudo-autosomal region. Scale bars: 5 µm.

(**B**) Immunostains using anti-FANCM and -SYCP3 antibodies in meiotic chromosome spreads from wild-type mice with and without FANCM peptide competition. Sex chromosomes are indicated by dashed squares. Consistent results were obtained with n = 3 independent wild-type mice. Scale bars: 5 µm.

(**C**) Representative immunostains of meiotic chromosome spreads in different stages of meiotic prophase using anti-FANCM, -FANCB, -FANCD2, -BRCA2, and -SLX4 antibodies as designated to the left of rows. Stages of meiotic prophase are labeled above columns. Scale bars: 5 µm.

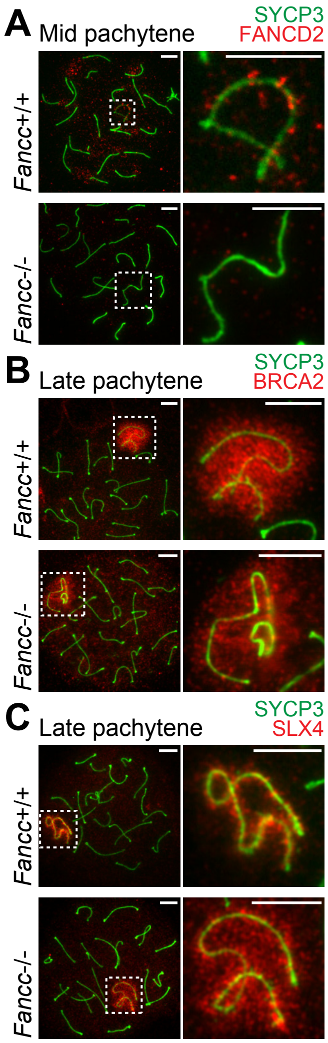

D *Fancc*-/-: Accumulation on the sex chromosomes

	EP	MP	LP	ED	LD
FANCD2					
BRCA2		domain	domain	domain	
SLX4	domain	domain	domain	domain	axial

present (green box)
absent (white box)

Figure S2. Function of FANCC in the FA-BRCA pathway during meiosis, related to Figure 2.

(**A-C**) Immunostains using indicated antibodies in meiotic chromosome spreads from *Fancc*-/- mice and wild-type littermate controls. Stages are labeled above, genotypes are labeled to the left. Dashed squares border sex chromosomes and are magnified to the right. Consistent results were obtained with n = 3 independent littermate pairs. Scale bars: 5 µm.

(**D**) Summary of temporal and spatial localization of anti-FA protein antibodies on the sex chromosomes in *Fancc*-/- mice; summaries of localization in wild-type mice are shown in Figures 1I and S1C.

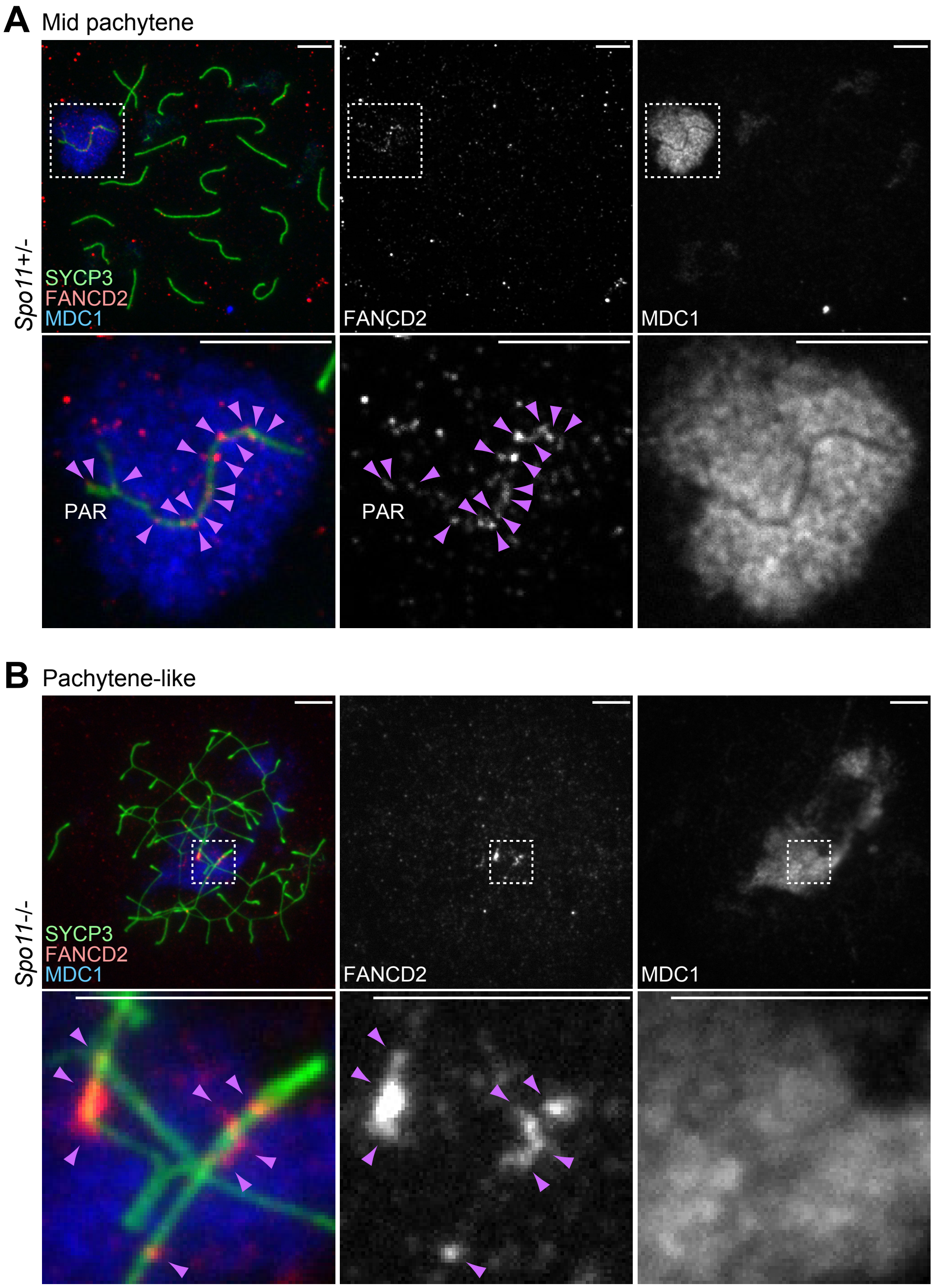

Figure S3. FANCD2 foci are present in the chromatin domain with meiotic silencing independent of SPO11-generated DSBs, related to Figure 5.

(**A, B**) Immunostains using indicated antibodies in meiotic chromosome spreads from *Spo11*-/- mice and control littermates. Stages are labeled above, genotypes are labeled to the left. Dashed boxes border selected nuclear regions and are magnified below. Arrowheads: colocalization of FANCD2 and MDC1. Consistent results were obtained with n = 3 independent littermate pairs. PAR: pseudo-autosomal region. Scale bars: 5 µm.

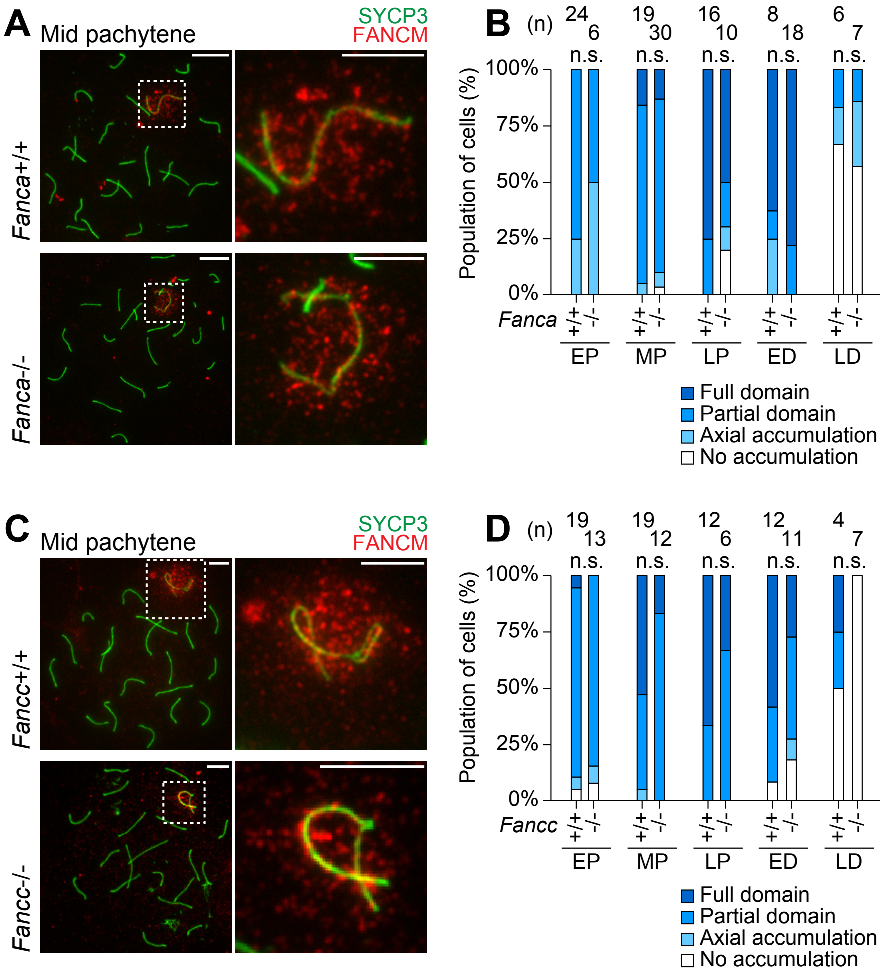

Figure S4. The FA core complex is dispensable for the regulation of FANCM, related to Figure 6.

(**A, C**) Immunostains using indicated antibodies in meiotic chromosome spreads from *Fanca*-/- (**A**) and *Fancc*-/- (**C**) mice, and corresponding wild-type littermate controls. Stages are labeled above, genotypes are labeled to the left. Dashed squares border sex chromosomes and are magnified to the right. Consistent results were obtained with n = 4 independent littermate pairs for each model. Scale bars: 5 µm.

(**B, D**) Categorical staining patterns for FANCM accumulation on sex chromosomes of *Fanca* (**B**) and *Fancc* (**D**) spermatocytes. Numbers of spermatocytes analyzed are noted above each graph. Accumulation was scored according to criteria described in the legend for Figure 4D. Data are aggregated from n = 3 littermate pairs for each model. p values are derived from Pearson's chi-square tests: n.s.: not significant.

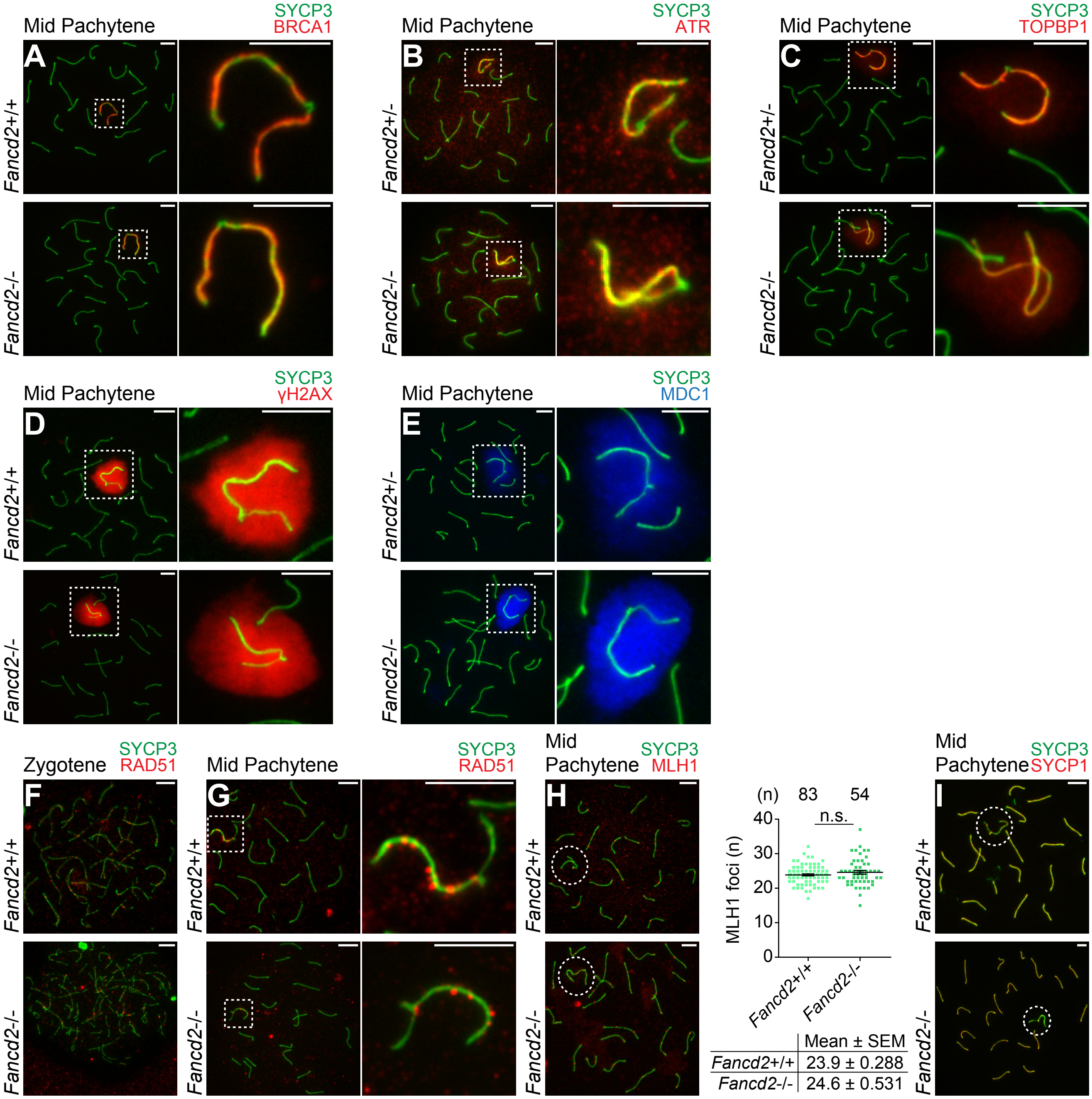

	Mean ± SEM
Fancd2+/+	23.9 ± 0.288
Fancd2-/-	24.6 ± 0.531

Figure S5. Regulation of DDR and recombination factors on the sex chromosomes in *Fancd2* mutant mice, related to Figure 6.

(**A-I**) Immunostains using the indicated antibodies in meiotic chromosome spreads from *Fancd2*-/- mice and wild-type or heterozygous littermate controls. Stages are labeled above, genotypes are labeled to the left. Dashed squares border sex chromosomes and are magnified to the right. Dashed circles border the sex chromosomes in (**H, I**). Consistent results were obtained with n = 3 independent littermate pairs. Scale bars: 5 µm.

(**H**) Bars represent means and SEMs. Mean ± SEM for each genotype is shown below. Data are aggregated from n = 3 littermate pairs of wild-type and mutant *Fancd2* mice. p values are derived from an unpaired, two-tailed Student's t test: n.s.: not significant, $p > 0.05$.

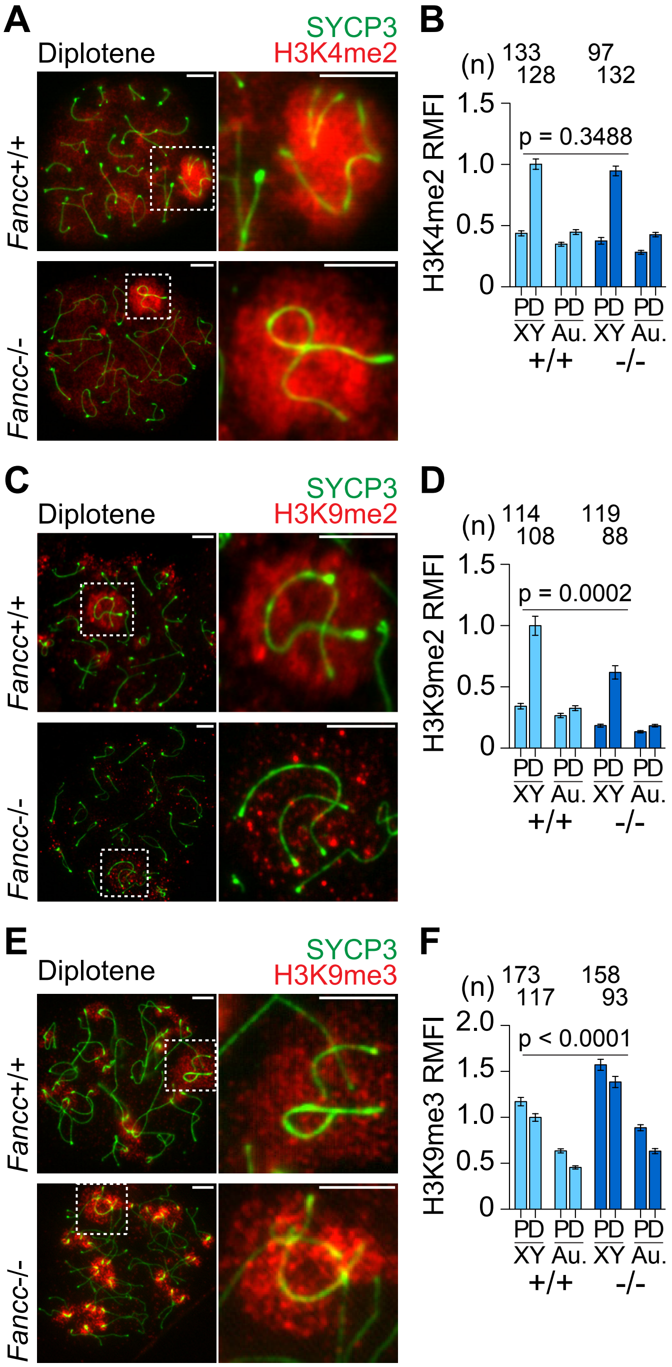

Figure S6. FANCC regulates H3K9 methylation, related to Figure 7.

(**A, C, E**) Immunostains using the indicated antibodies in meiotic chromosome spreads from *Fancc*-/- mice and corresponding wild-type controls. Stages are labeled above, genotypes are labeled to the left. Dashed squares border sex chromosomes and are magnified to the right. Scale bars: 5 µm.

(**B, D, F**) Quantification of H3K4me2 (**B**), H3K9me2 (**D**), and H3K9me3 (**F**) relative mean fluorescence intensity (RMFI) on sex chromosomes (XY) and autosome regions (Au.) in pachytene (P) and diplotene (D) spermatocytes. Numbers of spermatocytes analyzed are noted above each graph. Bars represent means and SEMs. Data are aggregated from n = 3 independent littermate pairs of wild-type and *Fancc*-/- mice. p values, indicated in the panels, are derived from one-way ANOVA and Tukey's method posttests.

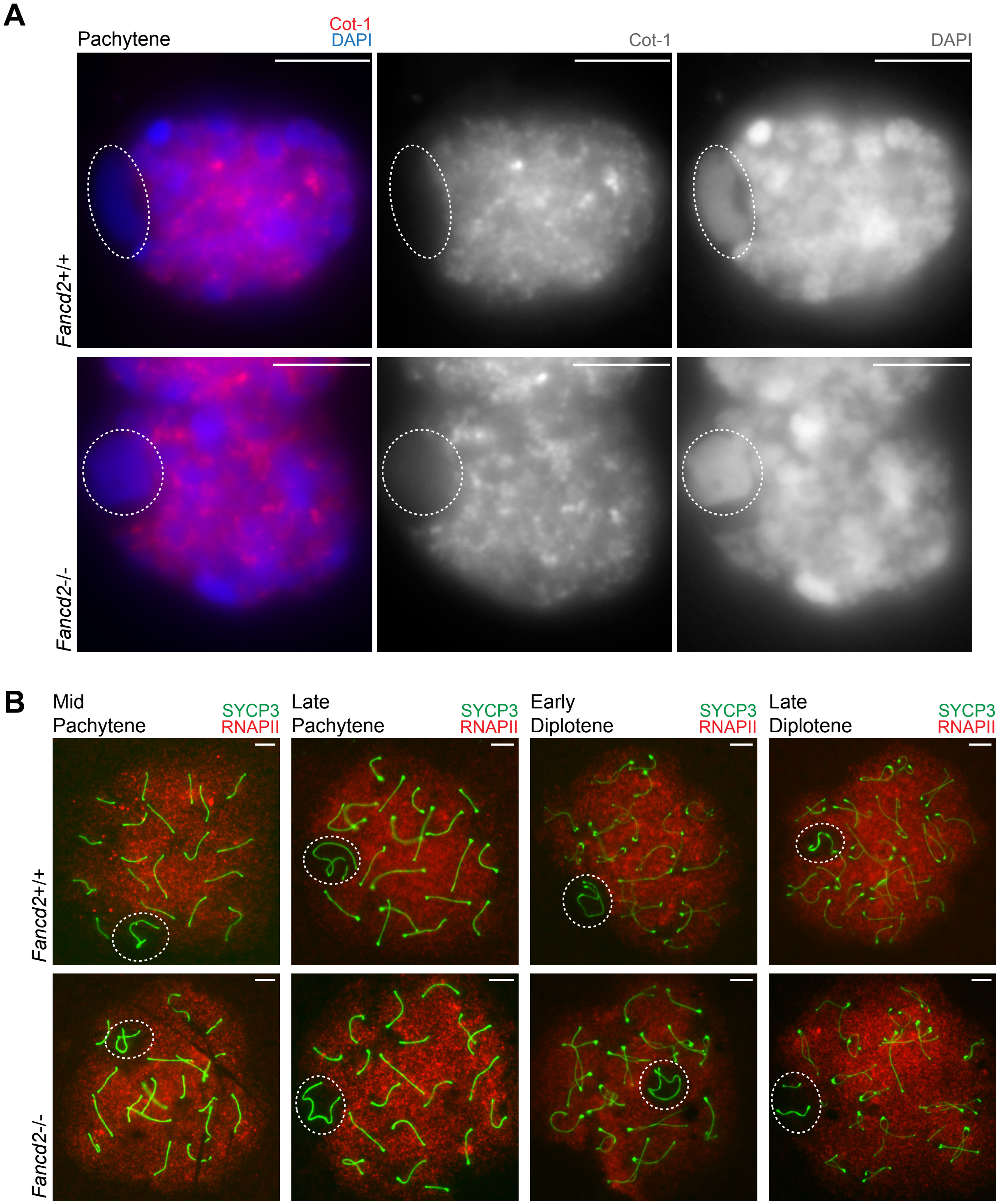

Figure S7. Large-scale detection of transcription remains unchanged in *Fancd2* mutants, related to Figure 7.

(**A**) Cot-1 RNA FISH (red signal) and DAPI (blue signal) in pachytene spermatocytes from *Fancd2*-/- and wild-type littermate control slides that maintain the three-dimensional architecture of nuclei (3D slides).

(**B**) Immunostaining of meiotic chromosome spreads using the indicated antibodies in *Fancd2*-/- mice and wild-type littermate controls.

(**A, B**) Consistent results were obtained with n = 3 independent littermate pairs of mice. Dashed circles border and denote the XY chromatin. Scale bars: 5 µm.

Supplemental Experimental Procedures

Generation of mice with conditional deletion of *Brca1* exon 11

Mice with conditional deletion of *Brca1* exon 11 using *Ddx4*-cre were previously described (Broering et al., 2014). Briefly, mice with floxed alleles for *Brca1* exon 11 (Xu et al., 1999) were obtained from the National Cancer Institute mouse repository. *Ddx4*-cre (also known as *Vasa*-cre) transgenic mice (Gallardo et al., 2007) were obtained from the Jackson Laboratory. Because the *Ddx4*-cre allele needs to be transmitted from the paternal allele to generate mice with a germline-specific conditional deletion, males with *Brca1*F/+ *Ddx4*-cre were mated with females homozygous for the floxed allele of *Brca1* exon 11 (*Brca1*F/F), and the conditional deletion model *Brca1*F/Δ *Ddx4*-cre (*Brca1*cKO) was obtained. We used *Brca1*F/+ *Ddx4*-cre littermates as controls.

Preparation and immunofluorescence of surface spreads of meiotic chromosomes

Meiotic chromosomes were analyzed with surface spreads prepared via hypotonic treatment, modified from an established protocol (Peters et al., 1997). Briefly, testes were excised and placed in PBS after removing extratesticular tissues. Seminiferous tubules (approximately one-quarter of an adult wild-type or heterozygous testis, and approximately one-half of an adult mutant testis) were transferred to a four-well dish (e.g., Thermo Scientific Nunc 4-Well Dishes, 144444) on ice. Three of the four wells contained 1 mL PBS, while the fourth well contained 1mL hypotonic extraction buffer (HEB, prepared as described in Peters et al.). In the first well containing 1 mL PBS, seminiferous tubules were gently unraveled into small clumps with fine-point tweezers, and care was taken not to tear or mince the seminiferous tubules. The clumps of seminiferous tubules were transferred to the second and third wells of 1 mL PBS for additional unraveling before transfer to the fourth well containing 1mL HEB. Once there, fine-point tweezers were used to carefully expose tubule surface area to HEB. The seminiferous tubules were incubated in HEB on ice for approximately three hours with gentle stirring every 30-45 minutes. After incubation, a small clump of seminiferous tubules—approximately four-to-six seminiferous tubules—was gently pulled and mashed between the tips of tweezers in 30 μL of sucrose (100 mM, pH 8.2) on a plain, uncharged microscope slide (e.g., Thermo Scientific Gold Seal, 3010-002). After approximately 15-25 mashes, a semi translucent cell suspension was formed. An additional 30 μL of sucrose was mixed with the suspension, gently pipetting up and down to mix and dilute the cell suspension. 30 μL volumes of the diluted cell suspension were applied to a positively charged slide (e.g., Thermo Scientific Probe On Plus, 22-230-900) that was incubated in chilled fixation solution (2% paraformaldehyde, 0.05% Triton X-100, and 0.02% sodium monododecyl sulfate, adjusted to pH 9.2 with sodium borate buffer, prepared as described in Peters et al.) for a minimum of two minutes. After applying the cell suspension/sucrose mixture, the slide was slowly, gently tilted up and down at slight angles to mix the cell suspension/sucrose mixture with remaining fixation solution. The previous steps were repeated until a desired number of slides were created. The slides were placed in closed humid chambers at room temperature for a minimum of one hour (maximum overnight) before opening the humid chamber lid to facilitate drying of the slides (approximately two hours). Once dry, the slides were washed in a low-concentration surfactant, 0.4% Photo-Flo 200 (Kodak, 146-4510), at room temperature two times for two minutes each. Slides were dried completely at room temperature (approximately 30 minutes) before staining or storage in slide boxes at -80°C.

For immunostaining experiments, surface spreads were incubated in PBST for 5-30 minutes before blocking in antibody dilution buffer (0.15% BSA, 0.1% Tween 20 in PBS), or 1% BSA dissolved in ddH2O, for an additional 30-60 minutes. Primary antibodies were diluted in antibody dilution buffer. Then, surface spreads were coated with 100 μL of the antibody solution, gently covered with Parafilm, and stored for a minimum of six hours (maximum overnight) in a

humid chamber at room temperature or 4°C. The following antibodies were used in this study: rabbit polyclonal anti-ATR (Cell Signaling, 2790), 1:50; rabbit polyclonal anti-BRCA1, 1:1500 (generated in the Namekawa lab (Ichijima et al., 2011)); rabbit polyclonal anti-BRCA2 (generated in the Andreassen lab in rabbits by fusing the 2800-3000 amino acid fragment of human BRCA2 to GST), 1:100; rabbit polyclonal anti-FANCB, 1:100 (generated in the Namekawa lab (Kato et al., 2015)); rabbit polyclonal anti-FANCD2 (E35), 1:200; rabbit polyclonal anti-FANCD2 (Novus, NB100-182), 1:200; rabbit polyclonal anti-FANCM (Fanconi Anemia Research Foundation, D3823), 1:100; rabbit polyclonal anti-H3K4me2 (EMD Millipore, 07-030), 1:500; mouse monoclonal anti-H3K9me2 (EMD Millipore, 07-441); rabbit polyclonal anti-H3K9me3 (EMD Millipore, 07-442), 1:250; rabbit polyclonal anti-RAD51 (Santa Cruz Biotechnology, sc-8349), 1:50; sheep polyclonal anti-MDC1 (Bio-Rad Antibodies, AHP799), 1:500; rabbit polyclonal anti-MLH1 (Santa Cruz Biotechnology, sc-11442), 1:100; mouse monoclonal anti-RNAPII (EMD Millipore, 05-952), 1:100; rabbit polyclonal anti-SLX4 (gift from Paula E. Cohen (Holloway et al., 2011)), 1:100; rabbit polyclonal anti-SYCP1 (Abcam, ab15090), 1:1500; mouse monoclonal anti-SYCP3 (Abcam, ab97642), 1:5,000; rabbit polyclonal anti-SYCP3 (Novus, NB300-231), 1:500; and rabbit polyclonal anti-TOPBP1 (gift from Junjie Chen (Yamane et al., 2002)), 1:500. After incubation of the primary antibodies, slides were washed three times for approximately five minutes each in PBST. Then, the slides were incubated with secondary antibodies conjugated to fluorophores (Thermo Fisher, Biotium, or Jackson ImmunoResearch). All secondary antibodies were diluted 1:500 in antibody dilution buffer. Slides were coated with 100 μL of antibody solution and then gently covered with Parafilm for approximately 30 minutes in humid chambers in darkness. Finally, slides were washed in PBST three times for five minutes each in darkness, then mounted in Vectashield (Vector Laboratories) containing 0.15% DAPI. Slides were either imaged immediately or stored at 4°C in darkness. For long-term storage, stained slides were kept at 4°C in darkness.

For double immunostaining using two primary antibodies from the same host species (rabbit polyclonal anti-FANCD2 antibody and rabbit polyclonal anti-RAD51 antibody), Fab fragments were used as suggested by the manufacturers of the secondary antibodies. Briefly, we performed immunostaining of anti-RAD51 antibody (six hours-to-overnight) and detected with Fab goat anti-rabbit IgG conjugated with Alexa Fluor 555 (Thermo Fisher). Then, slides were fixed in 1 mL of fresh, chilled 4% paraformaldehyde/1x PBS solution at room temperature for 10 minutes in a humid chamber. After briefly washing the fixed slides in PBST, we performed a second round of immunostaining with anti-FANCD2 antibody (six hours-to-overnight) followed by detection with Fab donkey anti-rabbit IgG conjugated with Alexa Fluor 647 (Thermo Fisher).

The specificity of FANCM antibodies was confirmed with peptide competition experiments using a FANCM peptide (CFDIQMLPNDLNQDRLKSDI) according to instructions at the Abcam website (http://www.abcam.com/protocols/blocking-with-immunizing-peptide-protocol-peptide-competition).

3D slide preparation and FISH

To conserve the morphology of meiotic chromatin, specialized slides that preserve the relative three-dimensional nuclear architecture of testicular germ cells were prepared as described (Namekawa, 2014; Namekawa and Lee, 2011; Namekawa et al., 2006). Briefly, seminiferous tubules underwent permeabilization, fixation, and then mechanical dissociation with fine-point tweezers before being cyto-spun onto positively-charged slides (e.g., Thermo Superfrost Plus, 12-550-15). Cot-1 RNA FISH was performed as described (Namekawa and Lee, 2011).

Microscopy and image analyses

All images of germ cells were acquired with an ECLIPSE Ti-E microscope (Nikon) and Zyla 5.5 sCMOS camera (Andor Technology), with 60x and 100x CFI Apochromat TIRF oil immersion lenses (Nikon), numerical aperture 1.40. Photoshop and Illustrator (CS6, Adobe) were used for composing figures. Primary spermatocytes were staged by staining for SYCP3 (described in detail in Fig. S1). For data analysis, the matched substage of meiotic prophase was analyzed in controls and mutants. All data were confirmed with at least three independent littermate pairs of mice.

Sample images of spermatocytes stained with anti-FANCD2, -BRCA2, -FANCM, and -MLH1 antibodies were blinded and manually scored with the ImageJ processing package Fiji (Schindelin et al., 2012). Sample images were blinded, scored, unblinded, and sorted through the following workflow: (1) Images were batch converted from the Nikon file format (.nd2) to the TIFF file format with ImageJ. (2) Composite TIFFs were batch blinded with ImageJ. (3) After focus counts were determined and recorded in Excel (Microsoft), the composite TIFF filenames were unblinded and sorted by genotypes and stages of meiotic prophase. (4) Data were imported to Prism 6 (GraphPad) for statistical analyses. Graphs of focus counts (FANCD2 and MLH1) were composed with Prism 6 and Illustrator; graphs of percentage accumulation (BRCA2 and FANCM) were composed with Excel and Illustrator.

H3K4me2, H3K9me2, and H3K9me3 signals were quantified with NIS-Elements Basic Research software (Nikon). Briefly, regions of interest (ROIs) were drawn around XY bodies, denoted as XY in Figs. 7 and S6, and prophase nuclei excluding the XY body, denoted as Au. (for "autosome region") in Figs. 7 and S6. The XY body and Au. ROIs were normalized to image background ROIs. For normalization of signals on a relative scale (0 to 1.5 for H3K4me2 and H3K9me2 analyses, 0 to 2 for H3K9me3 analyses), we calculated the mean of all diplotene XY body ROI signals and then divided all XY and Au. ROI signals by this value. This provided a relative value for ROI signals termed the "relative mean fluorescence intensity" (RMFI). The independent samples were combined and statistical analyses were run through Excel and Prism 6. RMFI graphs were composed with Prism 6 and Illustrator.

Cell culture
The human lymphoblast cell line PD20 (deficient for *FANCD2* (Timmers et al., 2001)) stably transduced with WT-FANCD2, the K561R mutant, or the empty pMMP retroviral vector (Li et al., 2010) were cultured in 10% fetal bovine serum RPMI 1640 medium containing 1 μg/mL puromycin. To induce monoubiquitination of FANCD2 in PD20 cells, DNA replication was arrested by treatment with 2 mM hydroxyurea, added from a 200 mM stock in growth medium kept at -20°C.

Western blotting
Whole testes and hydroxyurea-treated PD20 cells were dounce homogenized with RIPA buffer (10 mM Tris-HCl pH 7.5, 150 mM NaCl, 1 mM EDTA, 0.1% SDS, 0.1% NaDOC, 1% Triton X-100) containing protease inhibitor cOmplete (Roche). After the measurement of protein concentrations by the Bradford assay, lysates were mixed with an equal volume of 2x Laemmli sample buffer (prepared and stored at 4x concentration: 2% sodium dodecyl sulfate, 10% glycerol, 60 mM Tris-HCl pH 6.8, 1% v/v β-mercaptoethanol, and bromophenol blue), mixed with Benzonase (0.5 μL/100 μL lysate), and then incubated on ice for approximately 30 minutes, with gentle mixing every 10 minutes. If still viscous, the lysates were briefly sonicated on ice. Otherwise, the lysates were immediately boiled for 10 minutes. Using gels with 4% stacking and 6% running concentrations, SDS-PAGE was performed through 80 V in the stacking portion and 150 V in the running portion. Semi-dry transfer was performed with the Trans-Blot Turbo Transfer System (Bio-Rad, 1704155) by following the transfer method used by R&D Systems

(Bio-Techne; https://www.rndsystems.com/resources/protocols/western-blot-qc-protocol#Transfer). Using a PVDF membrane wetted in 100% methanol, semi-dry transfer was run for one hour with a constant current of 200 mA (15 V maximum). Afterwards, the membrane was blocked in StartingBlock Blocking Buffer (Thermo Scientific, 37538) for approximately 25 minutes, washed for 10 minutes in TBST two times, and then incubated with primary antibody. The following primary antibodies were diluted in TBST and then coated on the membrane for approximately one hour at room temperature: rabbit polyclonal anti-FANCD2 antibodies G33 (1:1000), E35 (1:1000), or Novus NB100-182 (1:1000). Then, the membrane was washed for 10 minutes in TBST three times, incubated in HRP-labeled anti-rabbit IgG diluted in TBST (1:5000), and incubated for one hour. After washing for 10 minutes in TBST four times, the membrane was visualized using Pierce ECL Western Blotting Substrate (Thermo Scientific, 32106) as instructed by the manufacturer.

References for this section

Broering, T.J., Alavattam, K.G., Sadreyev, R.I., Ichijima, Y., Kato, Y., Hasegawa, K., Camerini-Otero, R.D., Lee, J.T., Andreassen, P.R., and Namekawa, S.H. (2014). BRCA1 establishes DNA damage signaling and pericentric heterochromatin of the X chromosome in male meiosis. J Cell Biol *205*, 663-675.

Gallardo, T., Shirley, L., John, G.B., and Castrillon, D.H. (2007). Generation of a germ cell-specific mouse transgenic Cre line, Vasa-Cre. Genesis *45*, 413-417.

Holloway, J.K., Mohan, S., Balmus, G., Sun, X., Modzelewski, A., Borst, P.L., Freire, R., Weiss, R.S., and Cohen, P.E. (2011). Mammalian BTBD12 (SLX4) protects against genomic instability during mammalian spermatogenesis. PLoS Genet *7*, e1002094.

Ichijima, Y., Ichijima, M., Lou, Z., Nussenzweig, A., Camerini-Otero, R.D., Chen, J., Andreassen, P.R., and Namekawa, S.H. (2011). MDC1 directs chromosome-wide silencing of the sex chromosomes in male germ cells. Genes Dev *25*, 959-971.

Kato, Y., Alavattam, K.G., Sin, H.S., Meetei, A.R., Pang, Q., Andreassen, P.R., and Namekawa, S.H. (2015). FANCB is essential in the male germline and regulates H3K9 methylation on the sex chromosomes during meiosis. Human Molecular Genetics *24*, 5234-5249.!

Li, J., Du, W., Maynard, S., Andreassen, P.R., and Pang, Q. (2010). Oxidative stress-specific interaction between FANCD2 and FOXO3a. Blood *115*, 1545-1548.

Namekawa, S.H. (2014). Slide preparation method to preserve three-dimensional chromatin architecture of testicular germ cells. J Vis Exp, e50819.

Namekawa, S.H., and Lee, J.T. (2011). Detection of nascent RNA, single-copy DNA and protein localization by immunoFISH in mouse germ cells and preimplantation embryos. Nat Protoc *6*, 270-284.

Namekawa, S.H., Park, P.J., Zhang, L.F., Shima, J.E., McCarrey, J.R., Griswold, M.D., and Lee, J.T. (2006). Postmeiotic sex chromatin in the male germline of mice. Curr Biol *16*, 660-667.

Peters, A.H., Plug, A.W., van Vugt, M.J., and de Boer, P. (1997). A drying-down technique for the spreading of mammalian meiocytes from the male and female germline. Chromosome Res *5*, 66-68.

Schindelin, J., Arganda-Carreras, I., Frise, E., Kaynig, V., Longair, M., Pietzsch, T., Preibisch, S., Rueden, C., Saalfeld, S., Schmid, B*., et al.* (2012). Fiji: an open-source platform for biological-image analysis. Nature Methods *9*, 676-682.

Timmers, C., Taniguchi, T., Hejna, J., Reifsteck, C., Lucas, L., Bruun, D., Thayer, M., Cox, B., Olson, S., D'Andrea, A.D*., et al.* (2001). Positional cloning of a novel Fanconi anemia gene, FANCD2. Molecular Cell *7*, 241-248.

Xu, X., Wagner, K.U., Larson, D., Weaver, Z., Li, C., Ried, T., Hennighausen, L., Wynshaw-Boris, A., and Deng, C.X. (1999). Conditional mutation of Brca1 in mammary epithelial cells results in blunted ductal morphogenesis and tumour formation. Nat Genet *22*, 37-43.

Yamane, K., Wu, X., and Chen, J. (2002). A DNA damage-regulated BRCT-containing protein, TopBP1, is required for cell survival. Molecular and cellular biology *22*, 555-566.
